# Long noncoding RNA LINC01594 inhibits the CELF6-mediated splicing of oncogenic CD44 variants to promote colorectal cancer metastasis

**DOI:** 10.1038/s41419-023-05924-8

**Published:** 2023-07-14

**Authors:** Bowen Liu, Angxi Song, Pengkun Gui, Jin Wang, Yaojie Pan, Chao Li, Shuai Li, Yi Zhang, Tao Jiang, Yixin Xu, Dongsheng Pei, Jun Song

**Affiliations:** 1 Department of General Surgery, The Affiliated Hospital of Xuzhou Medical University. No. 99, Huaihai West Road, Quanshan District, Xuzhou, 221006 China; 2 Institute of Digestive Diseases, Xuzhou Medical University. No. 84, Huaihai West Road, Quanshan District, Xuzhou, 221002 China; 3 Department of Pathology, Xuzhou Medical University. No. 209, Tongshan Road, Yunlong District, Xuzhou, 221004 China; 4 Department of Medical Oncology, Zhejiang Provincial People’s Hospital. No. 158, Shangtang Road, Xiacheng District, Zhejiang, 310000 China

**Keywords:** Colon cancer, Cell invasion, DNA methylation, Long non-coding RNAs

## Abstract

Long noncoding RNAs (lncRNAs) play critical roles in tumorigenesis and tumor metastasis. However, the underlying mechanisms of lncRNAs in colorectal cancer (CRC) need further exploration. By using data from The Cancer Genome Atlas (TCGA) and GEO databases, we identified a novel CRC-related lncRNA, LINC01594, that is significantly upregulated in CRC and associated with poor prognosis. In vitro and in vivo, gain- and loss-of-function experiments demonstrated that LINC01594 promotes metastasis in CRC. LINC01594 functions as a DNMT1 scaffold, increasing the level of CELF6 promoter methylation. LINC01594 also competitively binds the transcription factor p53, decreasing CELF6 expression. This inhibited the exon skipping of CD44 V4–V7 induced by CELF6. In summary, this study highlights a novel CRC biomarker and therapeutic target, LINC01594, and the findings suggest that the LINC01594-CELF6-CD44 axis might serve as a biomarker and therapeutic target in CRC.

## Introduction

Colorectal cancer (CRC) accounts for ~10% of all cancer cases and cancer-related deaths worldwide [1]. Despite great progress in the treatment of CRC, ~25% of CRC patients present with metastatic disease at the time of initial diagnosis, and another 25% of patients diagnosed with early-stage CRC eventually develop metastasis [2, 3]. Overall, metastasis is responsible for most CRC-related deaths, and targeting CRC metastasis remains a challenge.

Long noncoding RNAs (lncRNAs) have gained increasing attention due to their emerging roles in gene regulation and contribute to a wide range of cellular functions, including development, differentiation, cell fate, and disease pathogenesis [4–6]. As a major member of the noncoding RNA (ncRNA) family, long ncRNAs (lncRNAs) have gained widespread attention. For instance, FLANC is highly expressed in metastatic CRC and promotes CRC metastasis and angiogenesis by prolonging the half-life of phosphorylated STAT3 [7]. In addition, LINC00941 promotes CRC metastasis by preventing SMAD4 protein degradation [8]. After activation by c-Myc, CASC11 targets heterogeneous ribonucleoprotein K (hnRNP-K) to stimulate WNT/β-catenin signaling in CRC cells [9]. In summary, some lncRNAs have been shown to contribute to CRC metastasis and could be used as biomarkers for cancer diagnostics and therapy.

Alternative splicing (AS) of messenger RNA (mRNA) produces a wide variety of differentially spliced RNA transcripts, which enrich the cellular protein reservoir and contribute to the temporal and spatial diversification of biological functions. As a new hallmark of cancers, AS is involved in multiple oncological processes, including angiogenesis [10], immune destruction [11], the regulation of cellular energetics [12], and especially invasion and metastasis [13]. The CUG-BP and ETR-3-like factor (CELF) RNA-binding protein family is a major class of splicing factors concentrated in the nucleus that play critical roles in AS. Alteration of CELF protein expression may lead to splicing events [14–16]. However, whether the CELF protein regulates AS in cancer metastasis remains to be further explored. Although a recent genome-wide transcriptome study showed that lncRNA-mediated AS outcomes affect multiple cellular signaling pathways that promote or suppress cancer progression [17], little is known about the function of lncRNA-mediated AS in CRC metastasis.

Here, we report that LINC01594 inhibits CELF6-mediated CD44 exon skipping through DNA methylation and competitive binding transcription factor and facilitates distant CRC metastasis in mice. These findings reveal a novel mechanism by which lncRNA-mediated AS induces CRC metastasis.

## Methods

### Clinical specimens

Between January 2012 and October 2014, 60 pairs of CRC tumor tissues and corresponding adjacent normal tissues were collected from patients with CRC who underwent surgery at Xuzhou Medical University Affiliated Hospital (Xuzhou, China). The patients did not receive any radiotherapy or chemotherapy before the operation. Diagnoses were pathologically confirmed, and the samples were immediately placed in liquid nitrogen after collection until use. The detailed clinicopathological features are summarized and analyzed in Additional file 1: Table S1. The study was conducted according to the guidelines of the Declaration of Helsinki. The research design, study protocols and information security were approved by the Ethics Committee of the affiliated hospital of Xuzhou Medical University (Xuzhou, Jiangsu, PR China), and written informed consent was obtained from all patients before enrolling in the research program.

### Cell culture and cell treatment

Human CRC cell lines (HCT116, SW620, SW480, DLD-1, LoVo) were purchased from the Cell Bank of the Chinese Academy of Science (Shanghai, China). The human normal colorectal epithelial cell line FHC was obtained from the American Type Culture Collection (Manassas, VA, USA). The cells were cultured in an appropriate medium supplemented with 10% fetal bovine serum (FBS; ExCell Bio, NY, USA) and 1% antibiotic/antimycotic solution and maintained in an incubator at 37 °C with 5% CO_2_ in a humidified atmosphere. DLD-1 and HT-29 cells were maintained in RPMI-1640 medium (Gibco, Thermo Fisher, Waltham, MA, USA).

### Cell transfection

Negative control siRNA (si-NC) and three individual LINC01594 siRNAs (si-LINC01594#1, #2, #3) were purchased from IBSBIO (Shanghai, China). CELF6 siRNA was purchased from Gene Pharma (Shanghai, China). The siRNA sequences are listed in Additional file 2: Table S2. Cells were transfected with siRNA at 30–50% confluence using **jetPRIME®** Polyplus Transfection (Polyplus-transfection S.A., Illkirch, France). LINC01594 and CELF6 were amplified from human cDNA as a template and cloned into the pcDNA3.1(+) vector (Invitrogen, USA). SW480, LoVo, and HCT116 cells were grown to 90% confluence before being transiently transfected with plasmids using Hieff Trans™ Liposomal Transfection Reagent (Yeasen Biotechnology, Shanghai, China) according to the manufacturer’s protocol. At 24–48 h posttransfection, cells were harvested for qRT-PCR or Western blot analysis.

### RNA extraction, reverse transcription-PCR, and qRT‒PCR

Total RNA from CRC tissues and cells was isolated and quantified using TRIzol reagent (Vazyme, Nanjing, China) according to the manufacturer’s instructions. LncRNAs and mRNAs were reverse transcribed using a SweScript RT I First Strand cDNA Synthesis Kit (Servicebio, Wuhan, China) in accordance with the manufacturer’s protocol. Relative quantification of LINC01594 and CELF6 was carried out by the 2-ΔΔCT method; 18S rRNA was used as an internal control when testing relative expression in tissues, and GAPDH was used as an internal control for cell lines. The reactions were performed independently in triplicate. Quantitative PCR assays were carried out with an ABI StepOne (Carlsbad, CA, USA) using 2 × SYBR Green qPCR Master Mix (High ROX) (Servicebio, China). The primer sequences are listed in Additional file 2: Table S2.

### Transwell assays

A modified two-chamber culture system with an 8-m pore size was used to test cell migration and invasion abilities using Transwell inserts (Corning Incorporated, USA) with or without Matrigel (BD Biosciences, USA) coating. Cells that had been transfected were plated into Transwell inserts, fixed with 4% paraformaldehyde solution and stained with 1% crystal violet after culture. We used an Olympus microscope to obtain images at a magnification of ×100 and ImageJ software to calculate the number of cells penetrating the pores. All experiments were carried out three times.

### Wound healing assay

Cells in incubation wells (1 × 10^6^) were wounded using a 100-µl plastic pipette tip. After 24 h of incubation, the wound sizes were assessed and imaged. The cell migration area between inscribed dashed lines in the wells was measured using ImageJ software (NIH, Bethesda) and normalized to control cells.

### Immunoblotting, immunofluorescence staining (IF), and antibodies

Cells were harvested with RIPA lysis buffer (Vicmed, China) supplemented with phenylmethylsulfonyl fluoride (PMSF). Lysates were cleared by centrifugation at 14,000 rpm for 15 min at 4 °C. The protein content of the cell lysates was quantified, and proteins were separated by SDS-polyacrylamide gel electrophoresis, transferred to a nitrocellulose filter membrane, blocked with 5% skim milk (BD Biosciences) in Tris-buffered saline with 0.05% Tween-20, and probed with specific antibodies. Chemistar™ High-sig ECL Western blotting Substrate (Tanon, Shanghai, China) was used to detect signals.

Cells grown on glass coverslips were fixed with 4% paraformaldehyde (PFA, Sigma Aldrich), permeabilized with 0.2% Triton X-100, and stained according to standard procedures. CoraLite488-conjugated goat anti-rabbit IgG (H + L) and CoraLite594-conjugated goat anti-rabbit IgG (H + L) were purchased from Proteintech (Wuhan, China). DAPI reagent was used to stain cell nuclei. The cells were imaged using an AXION SCOPE A1 HAL100 (ZEISS, Germany).

Antibodies against the following proteins were used: CELF6 (1:500, Proteintech), DNMT1 (1;2000, ABclonal), p53 (1:5000, Proteintech, China), E-cadherin (1:5000, Proteintech), and N-cadherin (1:5000, Proteintech).

### Tissue microarrays (TMAs) and RNA fluorescence in situ hybridization (RNA-FISH)

TMAs containing samples from 180 CRC patient tissues were purchased from Shanghai TUFEI Biotech Company. The Ethics Committee of Shanghai TUFEI Biotech Company approved this research. The RNA-fluorescence in situ hybridization (RNA-FISH) assay was accomplished by Servicebio (Wuhan, China). In brief, TMAs were fixed with 4% paraformaldehyde, trimmed, dehydrated, embedded, sectioned, stained, sealed and microscopically qualified in strict accordance with the SOPs for pathological laboratory examination of the Servicebio laboratory (Wuhan, China). A PANNORAMIC scanner (3DHISTECH, Hungary) was used to scan the TMAs. CaseViewer2.4 software (3DHISTECH, Hungary) was utilized for visualization, and Halo v3.0.311.314 software (Indica labs, U.S.A.) was used to calculate the positive rate (%) in the target area (positive cells/the total number of cells). The relationship between LINC01594 expression and clinicopathological features in TMA specimens is listed in Additional file 3: Table S3.

FAM-labeled LINC01594 probes were designed and synthesized by Servicebio (Wuhan, China) and were combined with target genes using a FISH kit (Servicebio, Wuhan, China) according to the manufacturer’s protocol.

### Methylation-specific PCR (MSP)

Cellular genomic DNA was isolated using a Genomic DNA extraction kit (TIANGEN, China), and the genomic DNA isolated was treated with bisulfite to convert unmethylated cytosine to uracil using a DNA Bisulfite Conversion Kit (TIANGEN, Beijing, China). The bisulfite-treated DNA was amplified using specific primers for methylated DNA (M-MSP) and unmethylated DNA (U-MSP) by PCR to determine CELF6 promoter methylation. The amplified DNA fragments were then subjected to 1.5% agarose gel electrophoresis, and images were taken. The MSP primers are listed in Supplemental Additional file 2: Table S2.

### RNA immunoprecipitation (RIP)

Cells were collected and resuspended in 2 ml of nucleus isolation buffer (1.28 M sucrose, 40 mM Tris-HCl pH 7.5, 20 mM MgCl_2_, 4% Triton X-100) and 6 ml of water for 20 min on ice. Next, 150 mM KCl, 25 mM Tris pH 7.4, 5 mM EDTA, 0.5 mM DTT, 0.5% NP40, 100 U/ml RNase inhibitor (ABclonal, Wuhan, China) and cocktail (Vicmed, Xuzhou, China) were added, and the nuclei were sonicated using an ultrasonic cell disruptor. Antibodies were added to the supernatant, which was rotated overnight at 4 °C. Magnetic beads were added for 2 h, and TRIZOL was used to purify the bead-bound RNA. RNA was detected by qRT-PCR.

### Chromatin immunoprecipitation (ChIP)

Cells were collected after formaldehyde crosslinking, resuspended in ChIP lysis buffer (50 mM Tris-HCl pH 8.0, 5 mM EDTA, 0.1% deoxycholate, 1% Triton X-100, 150 mM NaCl, 100:1 RNase inhibitor and cocktail added before use) and left on ice for 10 min. After ultrasonically shearing the cells, antibody coupling agent was added for 4 h. Subsequently, 25 µl of immunomagnetic beads was added, and incubation was continued for 1.5 hours. Chelex-100 was added, and the samples were boiled to release DNA. Then, 1 µl of proteinase K was added, and the samples were denatured at 55 °C at 1300 r/min. The samples were boiled for 10 min, 10 µl of linear polyacrylamide and 250 µl of anhydrous ethanol were added to the input group, and 10 µl of 3 M sodium acetate was added. The samples were placed at −80 °C for 30 min and centrifuged at 4 °C at 13,000 × *g* for 15 min. The precipitate was air-dried, and the supernatant was subjected to qRT-PCR detection.

### RNA pull-down assay

The RNA pull-down assay was carried out as follows. In brief, in vitro biotin-labeled LINC01594 was transcribed with Biotin RNA Labeling Mix (Roche Corporation, US) and T7 RNA polymerase (APExBIO, US), treated with RNase inhibitor, and purified with a clean-up kit (Promega Corporation, USA). The biotinylated LINC01594 probes were dissolved in binding and washing buffer and incubated with streptavidin agarose resin (Beyotime Biotechnology, China). Lysates of HCT116, SW480 and LoVo cells were incubated with probe-coated streptavidin beads, and products were separated by SDS‒PAGE.

### Chromatin isolation by RNA purification (ChIRP)

ChIRP-qPCR experiments were performed using published protocols [18] with slight modifications. The LINC01594 probes were designed by IBSBIO, and the sequences are listed in Additional file 2: Table S2. Briefly, 5 × 10^7^ SW480 cells were cross-linked with 1% formaldehyde for 10 min at RT and resuspended in lysis buffer (50 mM Tris HCl [pH 7], 10 mM EDTA, 1% SDS). and left on ice for 10 min. The lysate was sonicated using an ultrasonic cell disruptor (20 cycles, 30 s on/off) to obtain an average DNA size in the range from 200 to 600 bp and incubated with LINC01594 probes in hybridization buffer (500 mM NaCl, 1% SDS, 50 mM Tris–HCl, pH 7.0, 1 mM EDTA, 15% formamide, protease inhibitor cocktail, PMSF and RNase inhibitor) for 4 h at 4 °C. Subsequently, chromatin complexes were purified using magnetic streptavidin beads and then subjected to stringent washes, and DNA was eluted with a cocktail of 100 µg/ml RNase A (Beyotime, Shanghai, China) and 0.1 U/µl RNase H (Beyotime, Shanghai, China). The crosslinking was reversed by incubation in the presence of 0.1 µg/µl protease K, 150 mM NaCl, and 10 mM EDTA at 50 °C. DNA was then extracted with equal volumes of phenol, chloroform, and isoamyl and precipitated with ethanol at −80 °C overnight.

### Hematoxylin-eosin (HE) and immunochemical staining (IHC)

Sections of the CRC and adjacent tissue samples were deparaffinized in xylene and rehydrated with ethanol before paraffin embedding. All tissue samples were sectioned to produce 4-mm thick slices. To perform HE staining, the slices were stained with hematoxylin and eosin for 3 min and 5 s after dewaxing. For IHC, the slices were incubated in boiling 0.01 mol/l citric acid buffer for 15 min for antigen retrieval before incubation with primary antibodies. After incubation with the secondary antibody, DAB solution (Dako Denmark A/S) and hematoxylin were applied for staining before sealing.

### In vivo experiments

The animal studies were approved by the Institutional Animal Care and Use Committee (IACUC) of Xuzhou Medical University in Xuzhou, China. Mice were housed in a 12-h light/dark environment and had free access to a standard rodent diet and clean water. Male athymic 4-week-old BALB/c nude mice were obtained from the Animal Center of Gempharmatech Co., Ltd. (Nanjing, China) and maintained in a specific pathogen-free facility. For lung metastasis experiments, nude mice were randomly divided into two groups (*n* = 10 per group). HCT116 cells stably transfected with LINC01594 LV-RNA or scrambled control were harvested from six-well plates. CRC cells (2 × 10^6^) were administered via lateral tail vein injection (*n* = 10 per group). After 8 weeks, the mice were sacrificed, and the lungs were removed to observe lung metastases. The relative lung tissue anatomy was noted, and slices were used for further statistical analysis.

### Analysis of online datasets

The normalized lncRNA expression values in online-available datasets were downloaded from the TCGA database and GEO database (GSE84983, GSE110715 and GSE115856), and we first searched differentially expressed lncRNAs based on the TCGA dataset. We selected upregulated lncRNAs in tumor tissues according to the TCGA dataset. We then analyzed whether these selected lncRNAs were still upregulated in the GSE84983, GSE110715 and GSE115856 datasets. Finally, lncRNAs that were upregulated in these two datasets were identified. Q-PCR was used to determine the expression of lncRNAs in cell lines and tissues. Thus, the most differentially expressed lncRNA in CRC cell lines and tumor tissues was screened out. Therefore, LINC01594 was eventually selected. The heatmaps were determined according to the normalized expression levels of lncRNAs.

### Statistical analysis

GraphPad Prism 8.0.2 software (San Diego, CA, USA) was used to statistically analyze all data. Each experiments was repeated three times. Student’s t test was used when comparing differences between two groups. One-way ANOVA test was used to determain differences among more than two groups. Chi-square test or Fisher’s exact test were performed for categorical variables. Overall survivals (OS) analysis was used the Kaplan-Meier method. *p* < 0.05 (two-tailed) indicated statistically significant.

## Results

### LINC01594 is upregulated in CRC

**Fig. 1:**
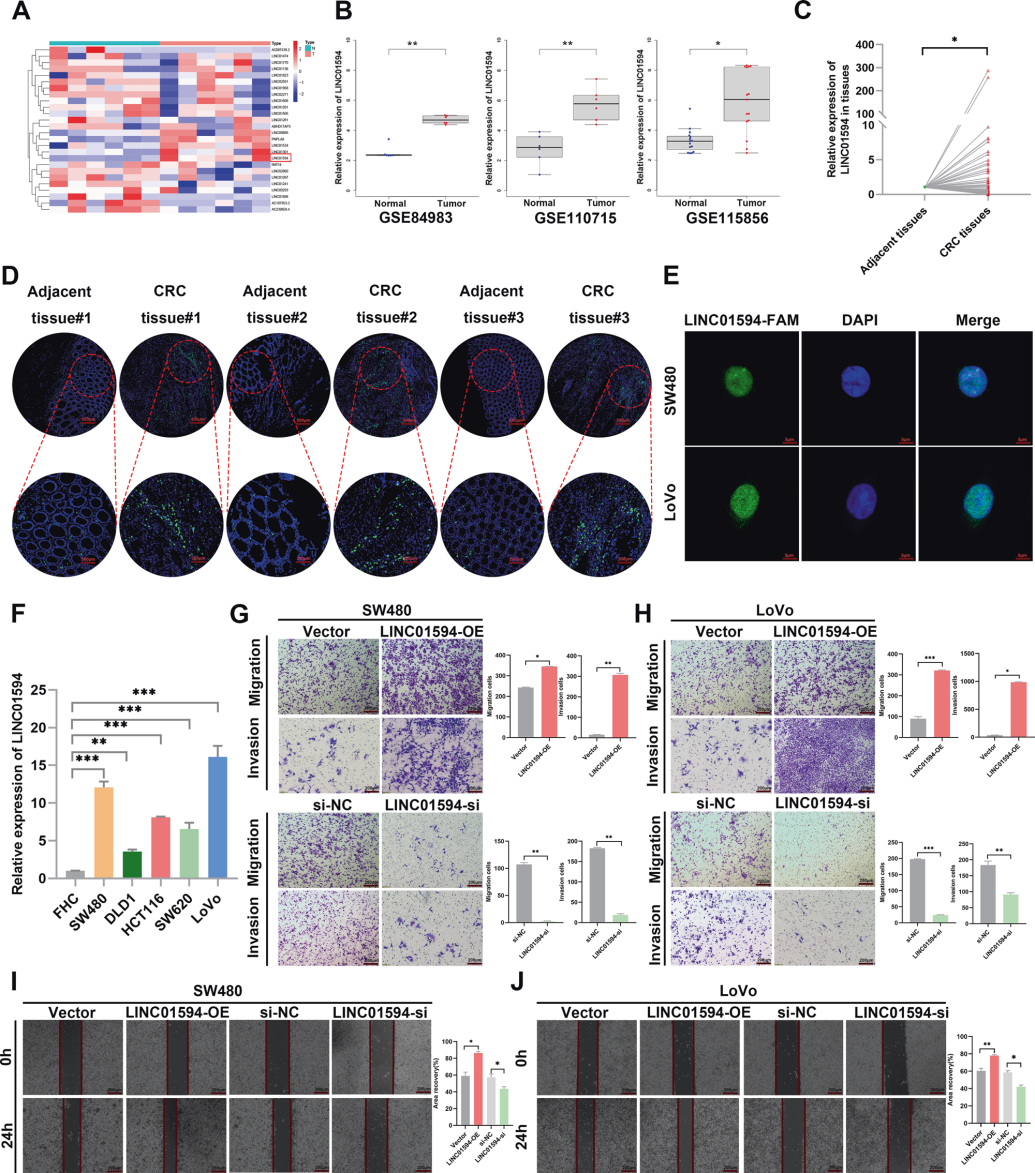
LINC01594 is significantly overexpressed in CRC tissues and is associated with poor prognosis. **A** Heatmap of abnormally expressed lncRNAs in CRC in the TCGA database. **B** LINC01594 expression in three GEO series studies (GSE84983, GSE110715, GSE115856). **C** The expression level of LINC01594 in 60 paired CRC tissues and nontumor specimens was determined by qRT-PCR. (*n* = 60). *P < 0.05. **D** FISH was used to determine the expression of LINC01594 in CRC TMAs. **E** FISH was performed to determine the subcellular distribution of LINC01594 in SW480 and LoVo cells. **F** The expression levels of LINC01594 in the normal human colon cell line FHC and five CRC cell lines (SW480, DLD1, HCT116, SW620 and LoVo) were examined using qRT-PCR. *P < 0.05, **P < 0.01 and ***P < 0.001. **G**, **H** Transwell assays were conducted to examine the effect of LINC01594 overexpression and knockdown on SW480 cell (**G**) and LoVo cell (**H**) migration and invasion. Statistical data are shown. (*n* = 3). *P < 0.05, **P < 0.01 and ***P < 0.001. **I**, **J** Wound-healing assays confirmed that LINC01594 promoted the metastasis of SW480 and LoVo cells. (*n* = 3). *P < 0.05, **P < 0.01 and ***P < 0.001.

To identify lncRNAs linked to CRC, The Cancer Genome Atlas (TCGA) (https://portal.gdc.cancer.gov/) was used to perform differential expression data analysis, which identified many abnormally expressed lncRNAs (Fig. 1A). Furthermore, we analyzed online datasets of lncRNA profiles in CRC tissues. We found that LINC01594 was also significantly upregulated in CRC in the GEPIA (http://gepia.cancer-pku.cn/) database as well as all three Gene Expression Omnibus (GEO) (https://www.ncbi.nlm.nih.gov/geo/) datasets (Additional file 4: Fig. S1A and Fig. 1B). To further verify these expression patterns in CRC tissues, the level of LINC01594 in 60 paired CRC tissues and adjacent normal tissues was detected (Fig. 1C). FISH staining assays with TMAs showed that LINC01594 was expressed at a high level in CRC (Fig. 1D and Additional file 4: Fig. S1B).

Thus, LINC01594 is significantly upregulated in CRC and may act as a biomarker. The full length of LINC01594 is 611 bp from NCBI (https://www.ncbi.nlm.nih.gov/). LINC01594 was expressed from chromosome 2 (Additional file 4: Fig. S1C). Furthermore, we determined that LINC01594 was primarily located in the nucleus through FISH experiments with SW480 and LoVo cells (Fig. 1E). Kaplan‒Meier survival analysis was performed to further determine whether the increase in LINC01594 in patients with CRC was associated with poor prognosis, and the results revealed that high levels of LINC01594 in our cohort were associated with decreased overall survival and disease-free survival (Additional file 4: Fig. S1D). To further analyze the relationship between LINC01594 expression and clinicopathological features, a chi-square test of TMAs and fresh-frozen CRC tissues was performed. We found that increased LINC01594 expression was positively associated with aggressive features of CRC, such as depth of invasion, lymph node metastasis, distant metastasis, and TNM stage progression, suggesting that LINC01594 plays an oncogenic role in driving the metastasis of CRC. However, there was no clear relationship between LINC01594 expression and tumor diameter, age and sex (Additional file 3: Table S3 and Table S4). Therefore, these results showed that LINC01594 may play an important role in the metastasis of CRC.

### Overexpression of LINC01594 promotes metastasis of CRC cells

QRT-PCR analysis was performed to detect LINC01594 expression in normal colonic epithelial FHC cells and CRC cells (SW480, HCT116, LoVo, DLD1 and SW620). LINC01594 expression was markedly higher in SW480, LoVo and HCT116 cells than in FHC cells (Fig. 1F). To determine the biological roles of LINC01594 in CRC cells, cells with knockdown or overexpression were established using siRNAs (si-LINC01594#38, si-LINC01594#147 and si-LINC01594#570) or one overexpression plasmid targeting LINC01594 and transfected into CRC cell lines (SW480 and LoVo) with the highest LINC01594 expression level to modify LINC01594 expression. The qRT-PCR results showed that the effect of si-LINC01594#38 and si-LINC01594#147 appeared to be weaker (Additional file 4: Fig. S1E) whereas the transfection of si-LINC01594#570 or OE-LINC01594 resulted in a significant change in the LINC01594 expression levels (Additional file 4: Fig. S1F). By performing Transwell assay, we demonstrated that interference with LINC01594 significantly inhibited the migration and invasion abilities of CRC cells, whereas the overexpression of LINC01594 promoted the migration and invasion abilities of CRC cells (Fig. 1G, H). Wound healing assay revealed that LINC01594 overexpression markedly facilited the migtation of CRC cells, whereas LINC01594 knockdown exerted the opposite effects (Fig. 1I, J). The expression level of LINC01594 was confirmed by qRT-PCR (Additional file 4: Fig. S1G).

Furthermore, a Western blot assay was conducted to detect epithelial-mesenchymal transition (EMT), and the results showed that LINC01594 promoted N-cadherin expression and inhibited E-cadherin expression (Additional file 4: Fig. S1H). Altogether, LINC01594 increased the capacity of CRC cells to metastasize in vitro.

### LINC01594 promotes CRC metastasis by inhibiting CELF6

**Fig. 2:**
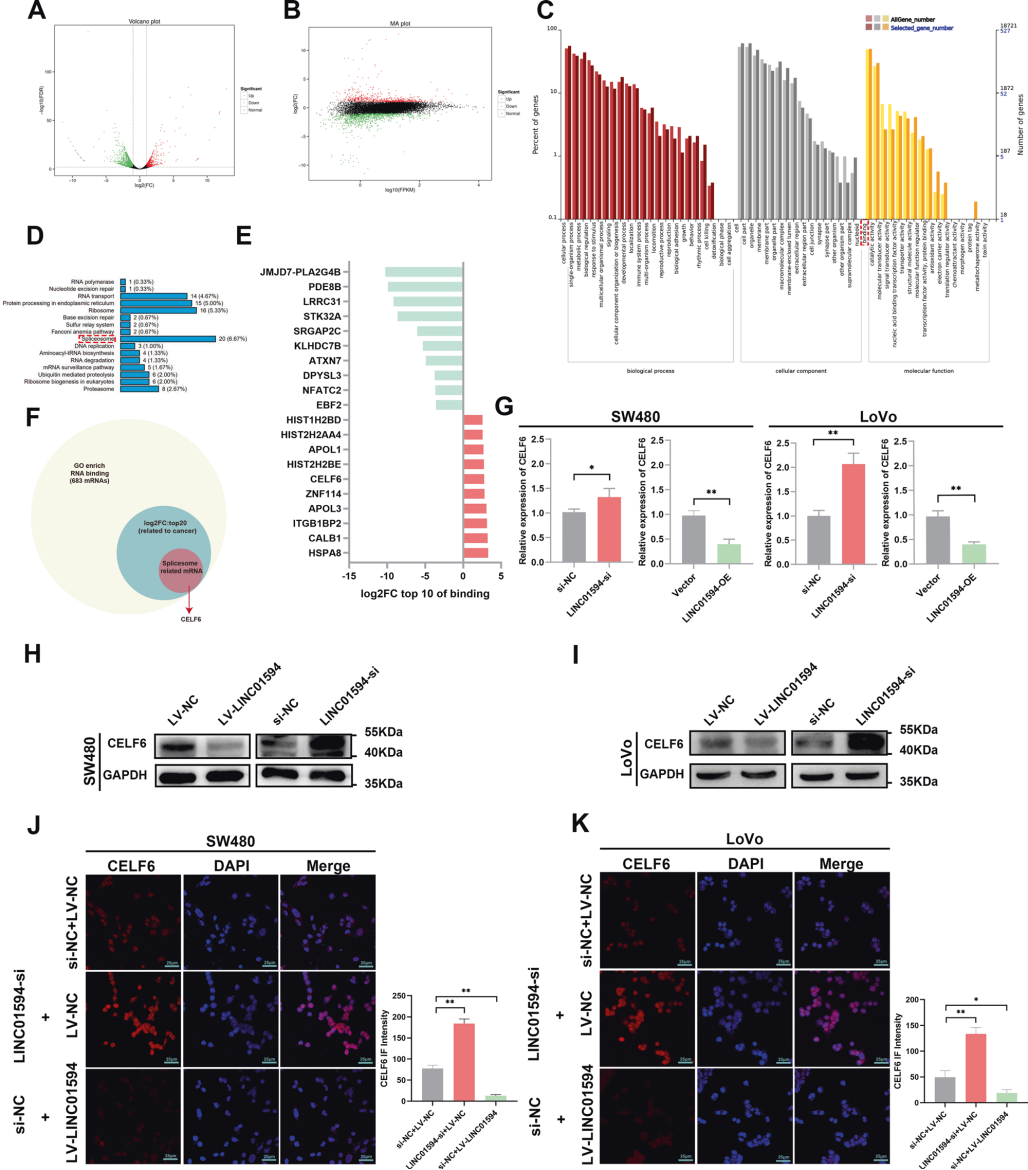
LINC01594 inhibits CELF6 expression. **A**, **B** The volcano map (**A**) and MA map (**B**) showed 1102 genes positively and 611 genes negatively associated with LINC01594 treatment. **C** GO classification map showing biological process cellular components and molecular functions associated with LINC01594. **D** KEGG classification map showing the most related genetic information processing with LINC01594. **E** Top 10 differentially expressed mRNAs positively and negatively correlated with LINC01594. **F** The top ten genes with positive and negative correlation differences with LINC01594 were taken from the GO annotated genes associated with binding. Among them, we identified the genes related to splicing function. **G** qRT‒PCR detected the mRNA level in SW480 and LoVo cells. (*n* = 3). *P < 0.05, **P < 0.01. **H**, **I** Western blotting was used to determine the levels of CELF6 in LINC01594-overexpressing and LINC01594-silenced SW480 cells (**H**) and LoVo cells (**I**). **J**, **K** IF assay showed that overexpression of LINC01594 inhibited CELF6 expression and knockdown of LINC01594 promoted CELF6 expression in SW480 cells (**J**) and LoVo cells (**K**). IF intensity statistical data are shown. (*n* = 3). *P < 0.05, **P < 0.01.

To further explore the mechanism of action of LINC01594 in CRC, we performed RNA-seq analysis of three pairs of SW480 cells transfected with si-NC and si-LINC01594. We found that when LINC01594 was downregulated, 1102 genes were upregulated and 4031 genes were downregulated (Fig. 2A, B).

Among all the genes that were regulated by LINC01594, multiple genes were related to the molecular function of binding according to the GO enrichment results (Fig. 2C), and the KEGG classification map showed the strongest correlation with spliceosomes (Fig. 2D). Hence, the top ten differentially upregulated and downregulated mRNAs among the GO enrichment results annotated as related to binding were selected (Fig. 2E) and screened for splicing-related genes, which revealed CELF6 (Fig. 2F). QRT‒PCR, Western blot and IF analyses confirmed that LINC01594 knockdown markedly upregulated the expression level of CELF6, whereas LINC01594 overexpression decreased the expression level of CELF6 (Fig. 2G–K) in CRC cells, and a correlation analysis of 8 pairs of tissues confirmed that LINC01594 was negatively associated with CELF6 (Additional file 4: Fig. S1I).

**Fig. 3:**
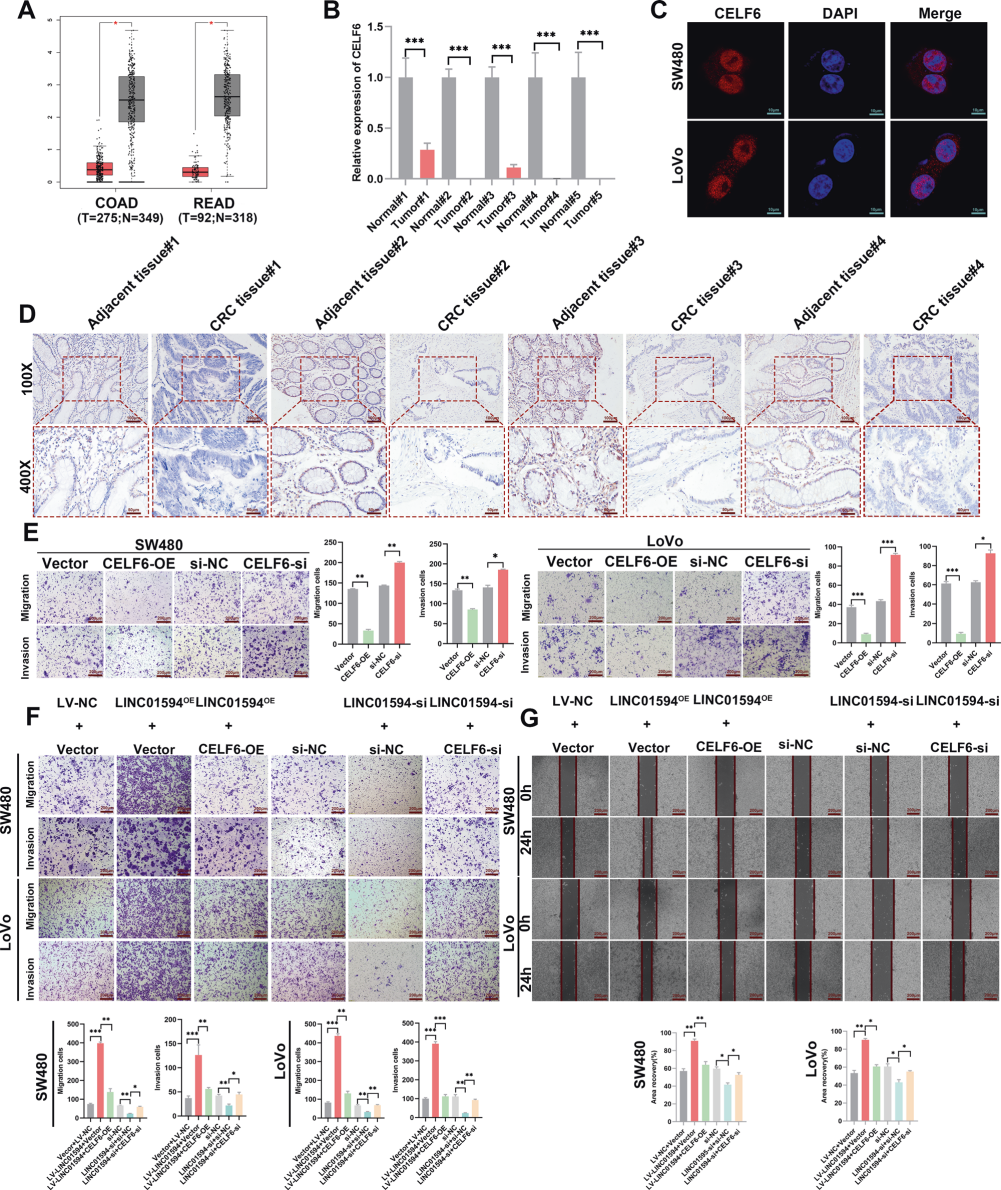
Low CELF6 expression in CRC and LINC01594 promote CRC metastasis by inhibiting CELF6. **A** The GEPIA online database showed that CELF6 was expressed at low levels in CRC (*p* < 0.05), and statistical data are shown. **B** qRT‒PCR was used to determine the expression of CELF6 in CRC. ***p < 0.001. **C** IF confirmed that CELF6 is located in the nucleus. **D** IHC was used to determine CELF6 expression in 4 pairs of adjacent tissues and CRC tissues. **E** Effect of CELF6 overexpression and knockdown on CRC cell migration and invasion. (*n* = 3). *p < 0.05, **p < 0.01, ***p < 0.001. **F** Effect of CELF6 re-expression and re-knockdown on the migration and invasion of SW480 and LoVo cells with LINC01594 overexpression and knockdown. (*n* = 3). *p < 0.05, **p < 0.01, ***p < 0.001. **G** Wound-healing assays of the effects of CELF6 re-expression and re-knockdown on the migration of SW480 and LoVo cells with LINC01594 overexpression and knockdown. (*n* = 3). *p < 0.05, **p < 0.01, ***p < 0.001.

CELF6 reportedly inhibits tumor progression [19, 20]. First, TCGA data showed that CELF6 was downregulated in multiple cancers, particularly in CRC (Additional file 4: Fig. S2A and Fig. 3A), and qRT‒PCR analysis further indicated significant downregulation of CELF6 expression in human CRC tissues (Fig. 3B). IF staining demonstrated that CELF6 localized to the nucleus (Fig. 3C). To further evaluate the role of CELF6 in CRC, we detected its expression in clinical CRC tissues by IHC, and CELF6 was significantly downregulated in CRC tissues relative to adjacent tissues (Fig. 3D). Transwell assays demonstrated that CELF6 inhibited the migration and invasion of CRC cells. Conversely, knockdown of CELF6 significantly enhanced these abilities (Fig. 3E), the expression level of CELF6 was detected by qRT-PCR (Additional file 4: Fig. S2B–C). These results suggest that CELF6 is involved in CRC inhibition.

To elucidate whether LINC01594 functions in CRC cells in a CELF6-mediated manner, we performed Transwell and wound healing assays. The results showed that the overexpression of LINC01594 promoted the metastatic potential of CRC cells, which was impaired by the simultaneous upregulation of CELF6. Conversely, the effect of LINC01594 knockdown on the invasion and migration of CRC cells was also partially attenuated by repression of CELF6 (Fig. 3F–G), the qRT-PCR results showed the expression level of LINC01594 and CELF6 (Additional file 4: Fig. S2D). Therefore, we hypothesized that LINC01594 facilitated tumor metastasis in a CELF6-mediated manner.

CELF6 belongs to the splicing factor CELF (1–6) family [21] and plays important roles in the post-transcriptional regulation of pre-mRNAs.

### LINC01594 represses CELF6 transcription by recruiting DNMT1 to the CELF6 promoter

LINC01594 is mainly expressed in the nucleus, and the function of lncRNAs is closely related to their subcellular localization. Given its nuclear localization, LINC01594 may interact with nuclear-specific proteins to regulate CELF6 expression [22–25].

**Fig. 4:**
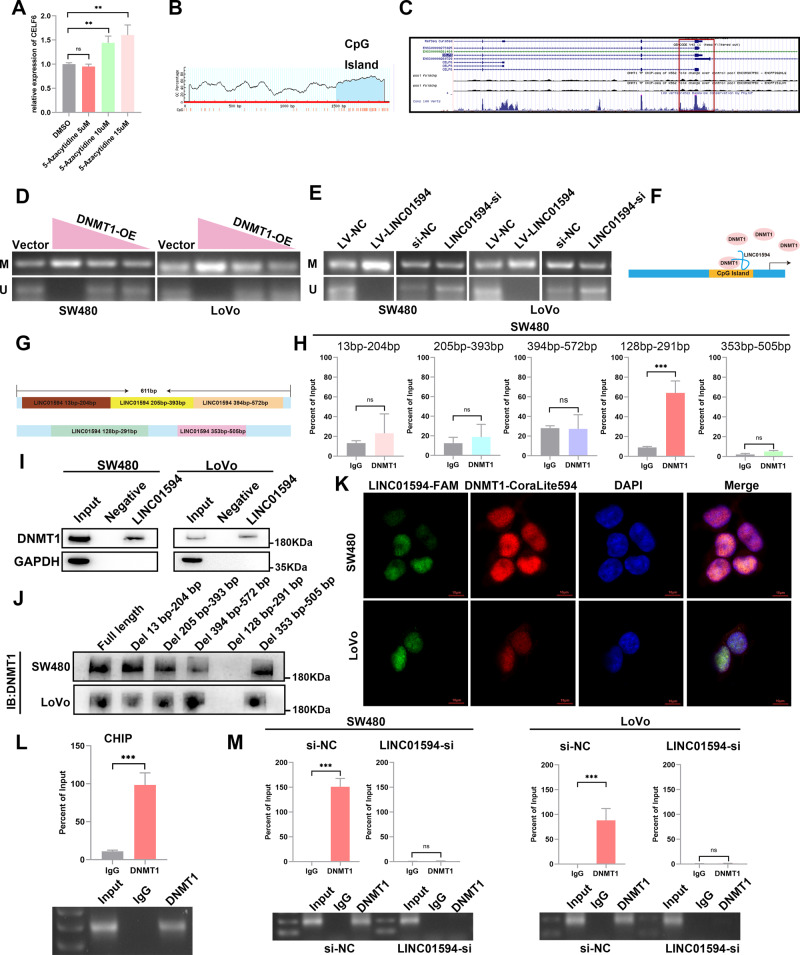
LINC01594 represses CELF6 transcription by recruiting DNMT1 to the CELF6 promoter region. **A** qRT‒PCR was used to determine CELF6 expression after 5-azacytidine treatment at 5, 10 and 15 µM. **p < 0.01. **B** The MethPrimer online database was used to predict the CpG island of the CELF6 promoter. **C** The ENCODE online database showed that DNMT1 interacted with the CELF6 promoter in the K562 cell line. **D** DNMT1 plasmids were transfected at 5, 3, and 1 µg into SW480 and LoVo cells. MSP assays were used to detect the methylation level of the CELF6 promoter. **E** MSP assays showed that LINC01594 promoted the methylation level of the CELF6 promoter. **F** LINC01594 recruits DNMT1 to the CpG island of the CELF6 promoter. **G** Five pairs of truncated primers were designed to detect 13–204 bp, 205–393 bp, 394–572 bp, 128–291 bp, and 353–505 bp of LINC01594. **H** RIP-qPCR verified that LINC01594 binds with DNMT1 in the 128–291 bp region in both SW480 cell lines. **I** RNA pulldown revealed that LINC01594 physically interacts with DNMT1 in vitro. **J** Western blotting of DNMT1 in samples precipitated by biotinylated full-length LINC01594 or LINC01594 truncations (deletion of 13–204 bp, deletion of 205–393 bp, deletion of 394–572 bp, deletion of 128–291 bp, and deletion of 353–505 bp). **K** FISH labeling of LINC01594 and immunofluorescence labeling of DNMT1 showed that LINC01594 and DNMT1 colocalize to the nucleus. **L** ChIP primers were designed in CpG islands, and ChIP‒qPCR showed that DNMT1 binds to CpG islands in the CELF6 promoter region. (*n* = 3). ***p < 0.001. **M** ChIP assays were performed after knocking down LINC01594, and the binding efficiency was measured by qRT‒PCR. (*n* = 3). ***p < 0.001.

Recently, an increasing amount of evidence has confirmed that lncRNAs interfere with the hypermethylation of target gene promoters and facilitate gene silencing [26, 27]. For example, MAGI2-AS3 promotes breast cancer by acting as a cis-acting regulatory element, and the DNA methylation level of MAGI2 is decreased in breast cancer [28]. Therefore, the increased methylation level of CpG islands might be the potential mechanism for CELF6 silencing. QRT‒PCR confirmed that the expression of CELF6 increased sequentially after 5-azacytidine treatment in a concentration-dependent manner (Fig. 4A). Thus, the downregulation of CELF6 in CRC might be associated with DNA methylation. We accordingly predicted and designed primers targeting CpG islands in the CELF6 promoter region (Fig. 4B) using the MethPrimer online database (http://www.urogene.org/methprimer/index.html). Using the ENCODE online database (https://www.encodeproject.org), DNMT1 was found to interact with the CELF6 promoter in the K562 cell line (Fig. 4C). Methylation-specific PCR (MSP) confirmed that DNMT1 regulates the methylation level of the CELF6 promoter (Fig. 4D) and revealed that the methylation level of the CELF6 promoter region was regulated by LINC01594 (Fig. 4E). Thus, the methylation of the CELF6 promoter is potentially mediated by DNMT1 and might be related to the aberrant expression of LINC01594 (Fig. 4F). Subsequently, RIP-qPCR was performed to validate the LINC01594-DNMT1 interaction. Five pairs of truncated primers were designed to detect the 13–204-bp, 205–393-bp, 394–572-bp, 128–291-bp, and 353–505-bp fragments of LINC01594 (Fig. 4G). RIP-qPCR verified that the 128–291-bp region of LINC01594 mediated its interaction with DNMT1 in CRC cells (Fig. 4H and Additional file 4: Fig. S2E). Biotinylated LINC01594 was transcribed, and RNA pulldown assays were performed to identify the proteins directly associated with LINC01594 in SW480 and LoVo cells (Fig. 4I). Subsequently, a series of deletion mutants were constructed based on the RIP assay (Additional file 4: Fig. S2F), particularly mutants within the region of LINC01594 containing 128 bp-291 bp, which mediated its physical interaction with DNMT1 (Fig. 4J) and failed to interact with DNMT1. Chromatin isolation by RNA purification (ChIRP) samples were used to amplify selected DNA regions by qRT-PCR (Additional file 4: Fig. S2G). By applying the ChIRP assay, we found that the CpG island of the CELF6 promoter can be precipitated by LINC01594 probes, which revealed the potential LINC01594 binding site on CELF6 promoter. Together, these findings indicate that LINC01594 interacts with DNMT1 and might be involved in DNMT1-mediated CELF6 silencing.

Moreover, FISH labeling of LINC01594 and immunofluorescence labeling of DNMT1 showed that LINC01594 and DNMT1 colocalize in the nucleus (Fig. 4K), and chromatin immunoprecipitation (ChIP) targeting CpG islands indicated that DNMT1 binds to CpG islands in the CELF6 promoter region (Fig. 4L). To further clarify this relationship, we performed ChIP assays again after knocking down LINC01594 and found that the binding efficiency of DNMT1 to the CELF6 promoter decreased relative to that in the control group (Fig. 4M). In summary, LINC01594 recruits DNMT1 as a scaffold to CpG islands in the CELF6 promoter region, increasing the methylation level of the promoter and suppressing CELF6 expression.

### p53 is a transcription factor of CELF6

**Fig. 5:**
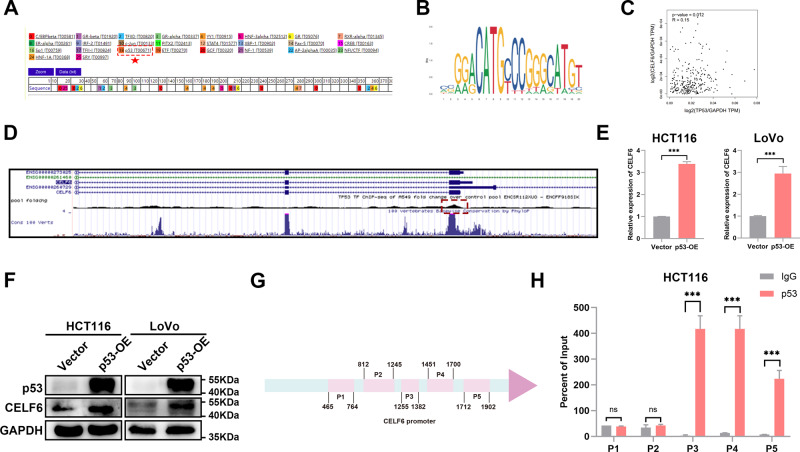
p53 is a transcription factor of CELF6. **A** Possible transcription factors of CELF6 were obtained from the PROMO online database. **B** The JASPAR database was used to predict the binding sites of p53 in the CELF6 promoter. **C** The GEPIA database showed that CELF6 was positively correlated with p53. **D** The ENCODE online database showed that p53 interacted with the CELF6 promoter in the A549 cell line. **E** Relative expression of CELF6 mRNA in p53-overexpressing CRC cells (HCT116, LoVo) compared with their respective controls measured by qRT‒PCR. (*n* = 3). ***p < 0.001. **F** CELF6 expression was detected in negative control and p53-overexpressing HCT116 or LoVo cells by Western blotting. **G** Five pairs of primers were developed for different regions of the ~2000 bp region upstream of the TSS denoted as P1–P5 to overlay the CELF6 proximal promoter region. **H** ChIP‒qPCR showed obvious enrichment of p53 in the P3-P5 region. (*n* = 3). ***p < 0.001.

In addition to the epigenetic regulation of targets in the nucleus, several studies have revealed that some lncRNAs can also regulate target genes by interacting with transcription factors. Therefore, to further investigate the roles of LINC01594 in CELF6 regulation, a detailed promoter analysis was performed to further explore the regulatory mechanisms underlying CELF6 expression. The sequence of the human CELF6 promoter was obtained from UCSC Genome Browser (http://genome.ucsc.edu/). Possible transcription factors of CELF6 were predicted using the PROMO online website. Interestingly, the database revealed that p53 might function as a potential transcription factor of CELF6 (Fig. 5A). Notably, multiple splicing landscapes are modulated by p53 in cancer [29–31]. The JASPAR website was then used to predict the binding site for p53 in the CELF6 promoter region (Fig. 5B). Pearson correlation analysis of CRC tissues showed that p53 expression was positively associated with CELF6 expression (Fig. 5C). Importantly, the results revealed specific binding of the transcription factor p53 at the promoters of CELF6 (Fig. 5D) using the ENCODE online database. It should be noted that p53 is mutated in SW480 cells; thus, in our assessment of p53, a p53 plasmid was transfected into HCT116 and LoVo cells to detect changes in CELF6 expression, and we found that CELF6 was upregulated by p53 (Fig. 5E, F). Next, to overlay the CELF6 proximal promoter region, 5 primers were designed for different regions of the ~2000-bp region upstream of the TSS denoted as P1-P5 (Fig. 5G), and p53 was observed to bind to the CELF6 promoter P3-P5 through ChIP assays (Fig. 5H). Together, these results demonstrated that p53 binds to the CELF6 promoter and facilitates the transcriptional activation of CELF6.

### LINC01594 competitively binds to p53 to repress CELF6 transcription

**Fig. 6:**
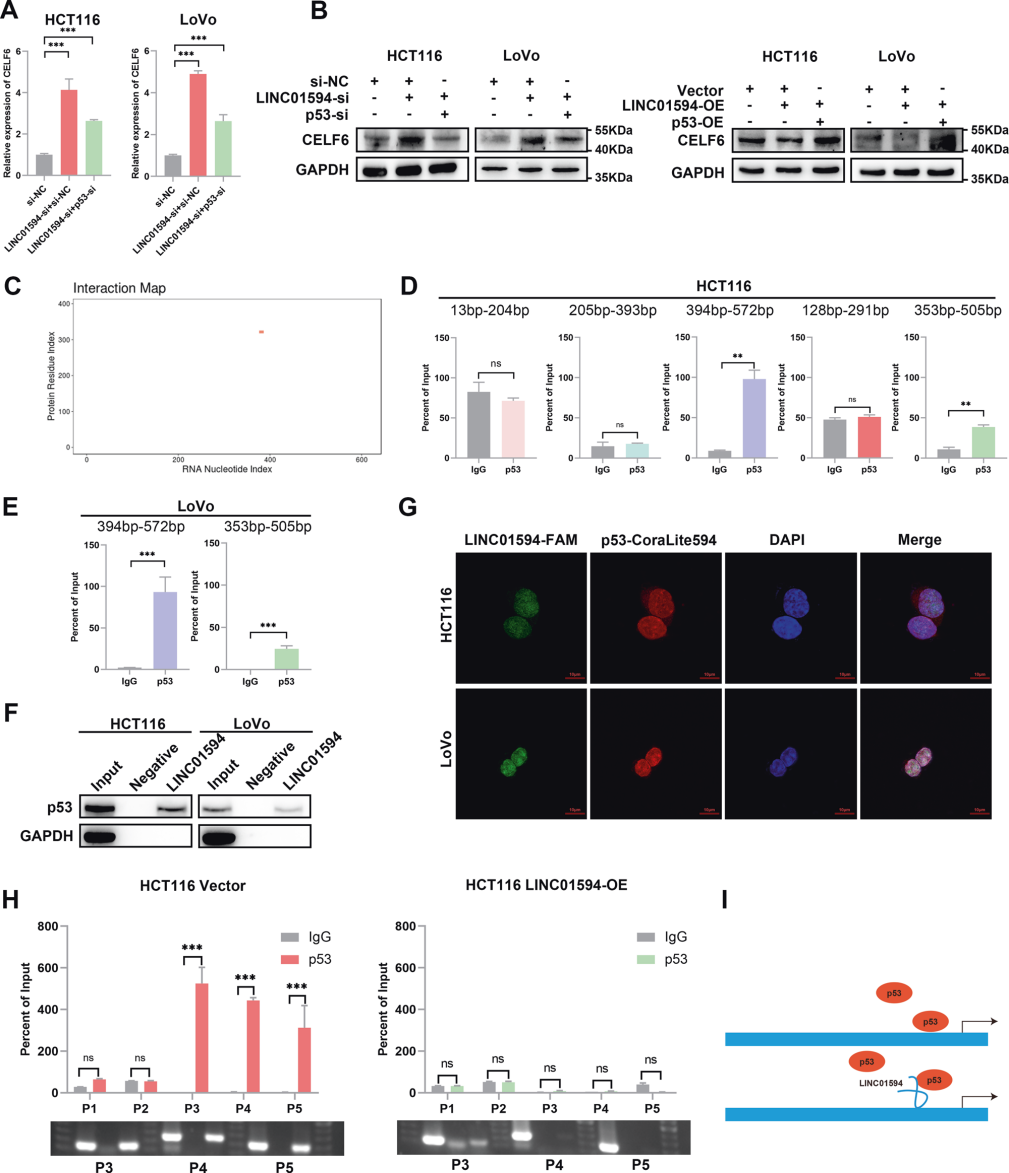
LINC01594 competitively binds to p53 to repress CELF6 transcription. **A**, **B** qRT‒PCR (**A**) and Western blot (**B**) showed that p53-mediated CELF6 overexpression in HCT116 and LoVo cells was reversed by LINC01594 overexpression. (*n* = 3). ***p < 0.001. **C** The CatRAPID online database was used to investigate the possible binding site between LINC01594 and p53. **D**, **E** RIP-qPCR verified that LINC01594 binds with p53 in the 394–572-bp region and 353–505-bp region in both SW480 and LoVo cells. (*n* = 3). **p < 0.01, ***p < 0.001. **F** RNA pulldown revealed that LINC01594 physically interacts with p53 in vitro. **G** FISH labeling of LINC01594 and immunofluorescence labeling of p53 showed that LINC01594 and p53 colocalize in the nucleus. **H** ChIP assays were performed after LINC01594 overexpression, and the binding efficiency was measured by qRT‒PCR. (*n* = 3). ***p < 0.001. **I** LINC01594 competitively binds to p53 to repress CELF6 transcription.

In addition, the knockdown of LINC01594 increased the expression of CELF6, which could be rescued by cotransfection of small interfering p53 (si-p53) (Fig. 6A, B). Taken together, these results suggest that p53 is a potential target by which LINC01594 inhibits CELF6 in CRC. Recently, several studies have reported that many lncRNAs regulate target genes by interacting with transcription factors. To investigate the underlying molecular mechanism by which LINC01594 regulates CELF6, the catRAPID algorithm was applied to analyze the potential binding sites of LINC01594 and p53 (Fig. 6C). Therefore, we hypothesized that LINC01594 might regulate CELF6 by interacting with p53. These binding sites were further verified by RIP-qPCR assay, and RNA pull-down experiments demonstrated that LINC01594 interacts with p53 in vitro (Fig. 6D–F). Consistent with the RIP-qPCR results, the LINC01594 deletion mutant lacking the binding domain (394–572 bp and 353–505 bp) failed to interact with p53 (Additional file 4: Fig. S3A). IF and FISH were employed to label p53 and LINC01594, respectively, and LINC01594 was found to colocalize with p53 in the nucleus (Fig. 6G). Furthermore, ChIP‒qPCR experiments demonstrated that the binding efficiency of p53 to the CELF6 promoter was reduced after the overexpression of LINC01594 (Fig. 6H). These data indicated that LINC01594 impairs p53 binding in the promoter regions and reduces the transcriptional activation of CELF6 (Fig. 6I).

### CD44 is a direct target of CELF6, and LINC01594 inhibits CD44 V4–V7 exon skipping by modulating CELF6

**Fig. 7:**
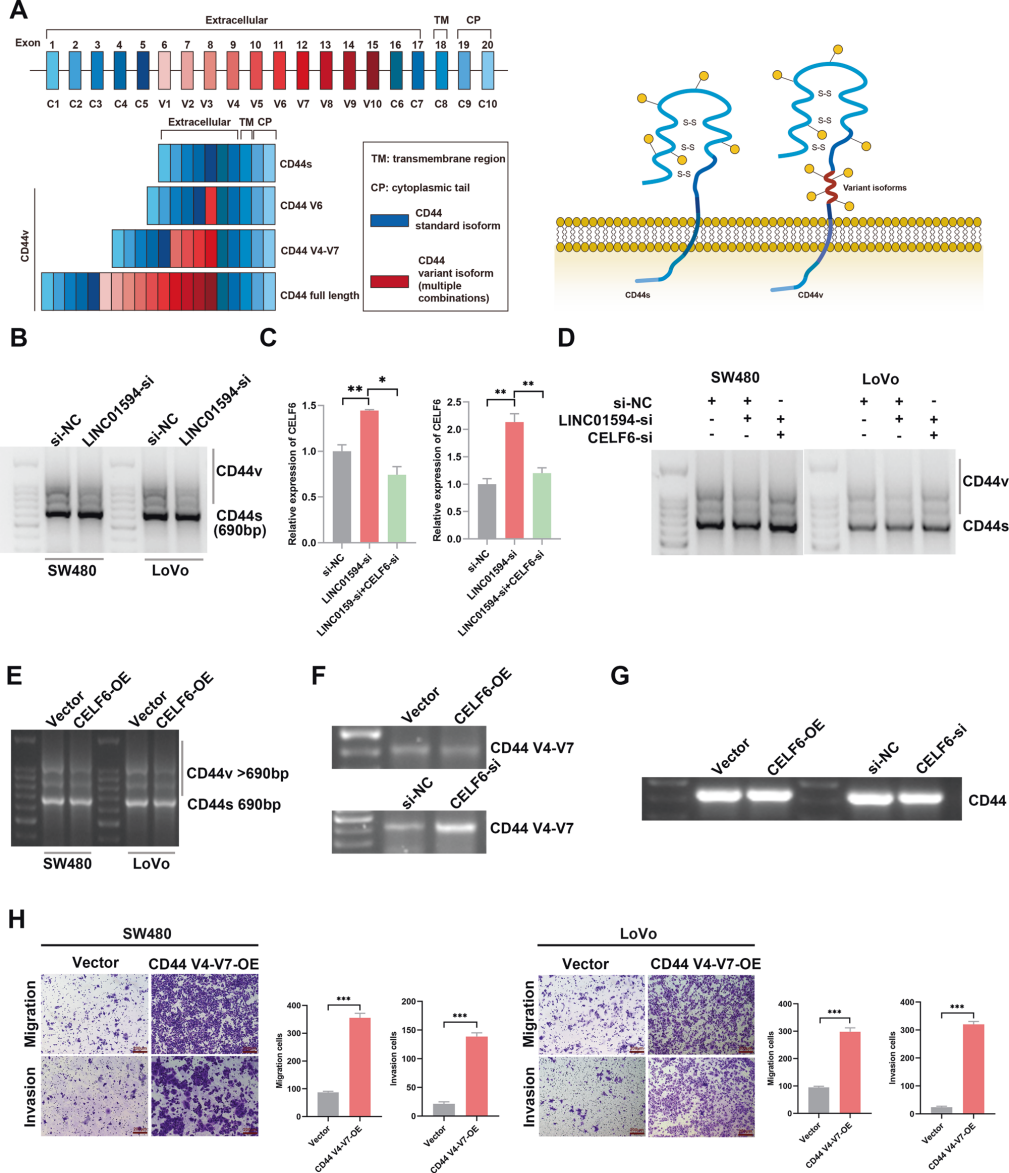
CD44 is a direct target of CELF6, and LINC01594 regulates CD44 V4–V7 exon skipping by modulating CELF6. **A** The CD44 protein is composed of an extracellular link domain, a stalk-like region in the extracellular domain close to the transmembrane region, where the variant exon products (red) are inserted, the transmembrane region (TM) and the cytoplasmic tail (CP). CD44v contains one or several variant regions (consisting of V1, V2, V3, V4, …V10), and various exons may constitute different CD44 variant isoforms via alternative splicing. **B** Representative images of semiquantitative PCR from HCT116 and LoVo cells following LINC01594 knockdown. The forward primer was designed on exon 2, and the reverse primers were designed on exon 15 and exon 16 of CD44 mRNA. The production of CD44s was 690 bp, and CD44v > 690 bp. **C** qRT‒PCR revealed that LINC01594-mediated CELF6 expression was reversed by CELF6 knockdown. (*n* = 3). *p < 0.05, **p < 0.01. **D** Rescue tests showed that knockdown of LINC01594 and CELF6 reversed the loss in CD44v expression induced by LINC01594 knockdown alone. **E** Semiquantitative PCR tested the expression level of CD44v isoforms in SW480 and LoVo cells with CELF6 overexpression. **F** Primers were designed to detect CD44 v4–v7 expression in CELF6-overexpressing and CELF6-silenced SW480 and LoVo cells. The forward primer was designed on variant exon 4 (v4), and the reverse primer was designed on variant exon 7 (v7). The production length was 429 bp. **G** The CD44 forward primer was designed on exon 15 and exon 16, and the reverse primer was designed on exon 18 to determine the expression of the CD44 standard isoform. **H** Transwell assays were conducted to examine the effect of CD44 v4–v7 isoform overexpression and knockdown on SW480 and LoVo cells. (*n* = 3). ***p < 0.001.

Interestingly, the gene with the most significant splicing changes after CELF6 depletion was CD44 in lung cancer [21]. CD44 is a cell adhesion protein that is expressed in multiple isoforms from a pre-mRNA comprising 20 exons. Typically, the first and last five exons are retained in the mature CD44 mRNA to form the CD44s (standard) isoform. CD44 variant isoforms (CD44v) are generated by inclusion of some of the ten remaining exons (Fig. 7A). Importantly, alternative splicing of CD44 is common in CRC, and CD44v is substantially expressed in metastatic CRC [32].

Subsequently, CD44v primers were designed (Additional file 4: Fig. S4B). We knocked down LINC01594 and observed reduced expression of CD44v (Fig. 7B), and the depletion of LINC01594 resulted in more frequent exclusion of CD44 alternative exons. Rescue tests showed that the knockdown of LINC01594 and CELF6 reversed the loss in CD44v expression induced by LINC01594 knockdown alone (Fig. 7C, D). To further clarify the relevance of CELF6 and alternative splicing of CD44, we also tested whether the overexpression of CELF6 would result in the silencing of CD44v isoforms, the result showed that overexpression of CELF6 inhibited the expression of CD44v (Fig. 7E). Overall, the knockdown of CELF6 promotes the expression of CD44 oncogenic isoforms.

It has been reported that a CD44 variant isoform covering the V4–V7 exon initiates metastasis formation of a locally growing tumor in a rat tumor model [33], and stable CD44v4-v7 knockdown in a highly metastatic tumor line revealed a striking reduction in the metastatic capacity [34]. CD44 V4–V7 was detected to explore the variant region regulated by CELF6 (Additional file 4: Fig. S4C). RT‒PCR and agarose gel electrophoresis revealed that the CD44 V4–V7 region was inhibited by CELF6 (Fig. 7F). Assessment of the standard CD44 isoform showed that CELF6 promotes CD44 V4–V7 exon skipping instead of suppressing CD44 expression (Fig. 7G and Additional file 4: Fig. S4D). A CD44 V4–V7 plasmid was transfected into SW480 and LoVo cells, and Transwell assays validated the promotion of CRC metastasis (Fig. 7H). Together, these data collectively uncover a previously unrecognized role of LINC01594 in the regulation of alternative splicing and reveal a growth-promoting function of the LINC01594-CELF6-CD44 regulatory axis in CRC cells.

### LINC01594 promotes the in vivo dissemination of CRC cells

**Fig. 8:**
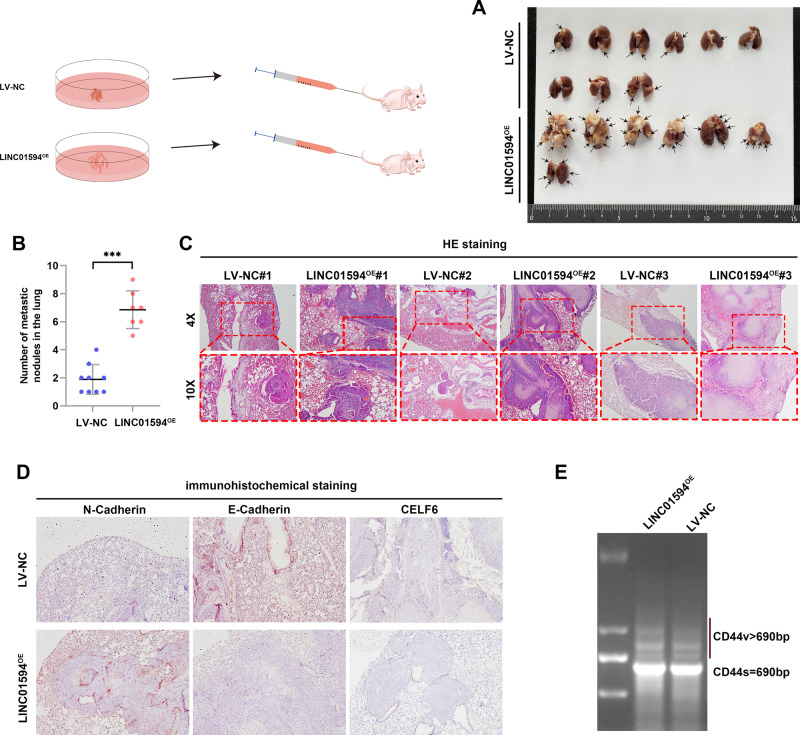
LINC01594 promotes in vivo dissemination of CRC cells. **A** A total of 5 × 10^6^ HCT116 lentivirus-NC (LV-NC) and LINC01594 stable‐overexpression (LINC01594^OE^) were injected via the lateral tail vein. Lung images at week 6 are shown. **B** Statistical analysis of lung metastasis foci. ***p < 0.001. **C** HE-stained lung sections are shown. **D** IHC assays showed the lung metastasis of LINC01594^OE^-injected mice exhibited lower expression of CELF6 and E-cadherin and higher expression of N-cadherin. **E** Agarose gel electrophoresis was used to show that the expression level of CD44v was significantly higher in the LINC01594^OE^ group.

Given that migration and invasion are essential for cancer metastasis, we investigated the role of LINC01594 in CRC metastasis in vivo. We injected HCT116 cells with lentivirus-induced stable overexpression of LINC01594 and negative control into the tail veins of 10 pairs of mice, and 6 weeks later, we observed that significantly more metastases formed in the lungs of mice injected with LINC01594^OE^ than in those of mice injected with LV-NC (Fig. 8A, B). HE staining demonstrated more metastatic foci in the lungs of the LINC01594^OE^ group than in the lungs of the LV-NC group (Fig. 8C).

In addition, the lung metastasis of LINC01594^OE^-injected mice exhibited lower expression of CELF6 and E-cadherin and higher expression of N-cadherin (Fig. 8D). We further performed CD44 RT‒PCR to determine whether the CD44 variant exon was skipped in the lung metastasis model (Fig. 8E), and the expression level of CD44v was significantly higher in the LINC01594^OE^ group. Collectively, these findings indicate that LINC01594-mediated oncogenic CD44 variant overexpression promotes CRC metastasis to distant organs in mice.

## Discussion

An increasing number of studies have revealed a key regulatory role for lncRNAs in the carcinogenesis and progression of human cancers [35]. However, the potential biological functions and underlying mechanisms of most lncRNAs in human CRC remain elusive. In the present study, the top twenty upregulated lncRNAs were filtered from the TCGA database, and LINC01594 was found to be markedly overexpressed in CRC tissues compared to nontumorous tissues. We are the first to demonstrate by loss-of-function and gain-of-function assays that LINC01594 may function as a prometastatic factor in CRC.

According to estimates, alternative splicing generates splice variants for more than 90% of human genes [36, 37], and 60% of these variants are translated into distinct protein isoforms [38]. Competition between splicing factors that promote exon inclusion and those that support exon skipping determines the overall function of these splicing factors [39–41]. Previous reports indicate that the elusive expression of splicing factors could be mediated by upstream genes in CRC [30, 42, 43]. Moreover, we found that LINC01594 inhibits CELF6 expression and thus enhances CD44 V4–V7 expression. The CELF6 protein was first identified in yeast as a splicing factor of exon skipping in muscle [16], and it usually plays an important role in mRNA stability in cancer. For instance, CELF6 stabilizes p21 mRNA to control cancer cell proliferation [19]. Consistent with recent research, CELF6 stabilizes FBP1 by binding to its 3’UTR to modulate triple-negative breast cancer progression [20]. In contrast, we confirmed that CELF6 modulates alternative splicing of CD44v during CRC metastasis, and CD44v has been reported to promote CRC progression [30, 44], especially metastasis. Furthermore, CELF6 has been confirmed to inhibit CD44 V4–V7 expression. The roles of CD44 variant isoforms other than CD44 V4–V7 in cancer metastasis have been confirmed [45, 46]. However, there are scant reports of CD44 V4–V7 since it was reported to confer metastatic potential in nonmetastatic pancreatic cancer in rats two decades ago [33, 47]. The present study provides evidence that CD44 V4–V7 promotes CRC metastasis. Interestingly, Keiichiro Sakuma et al. reported that CD44 V6 promotes tumor metastasis [32, 48], in line with our data. In clear contrast, upregulation of CD44s attenuates breast cancer metastasis and stemness [49, 50]. For example, Keiichiro Sakuma et al. stated that the link between the CD44 variant isoform and tumor metastasis may differ among cancers of different origins or between normal cells and cancer cells [32]. This study is the first to demonstrate that CELF6 negatively regulates CRC metastasis by controlling alternative splicing of CD44. Nevertheless, it is possible that downregulation of CELF6 contributes to CRC progression through other unidentified mechanisms.

Studies in the last two decades have focused on lncRNAs potentially impacting the expression levels of miRNA targets by competing for miRNAs [51]. It has been suggested that lncRNAs regulate cell signaling by functioning as scaffolds that interact with various signaling molecules. For example, lncRNA GClnc1 acts as a modular scaffold of WDR5 and KAT_2_A complexes to specify histone modification patterns [52]. In the present study, LINC01594 was found to function as a molecular scaffold to promote DNMT1 binding to CpG islands, enhance the DNA methylation level of the CELF6 promoter and inhibit transcription. In addition, growing evidence suggests that a large number of transcription factors interact with lncRNAs, and these interactions could play an important role in their regulation [53, 54]. For instance, lncRNA DSCAM-AS1 enhances the recruitment of YBX1 to the promoter regions of FOXA1 and Erα [55]. Here, we show that LINC01594 competitively binds with p53 to inhibit the transcription of CELF6. The detection of LINC01594 enrichment at the CELF6 promoter by CHRIP supports the notion that LINC01594 participates in the regulation of CELF6 transcription.

**Fig. 9:**
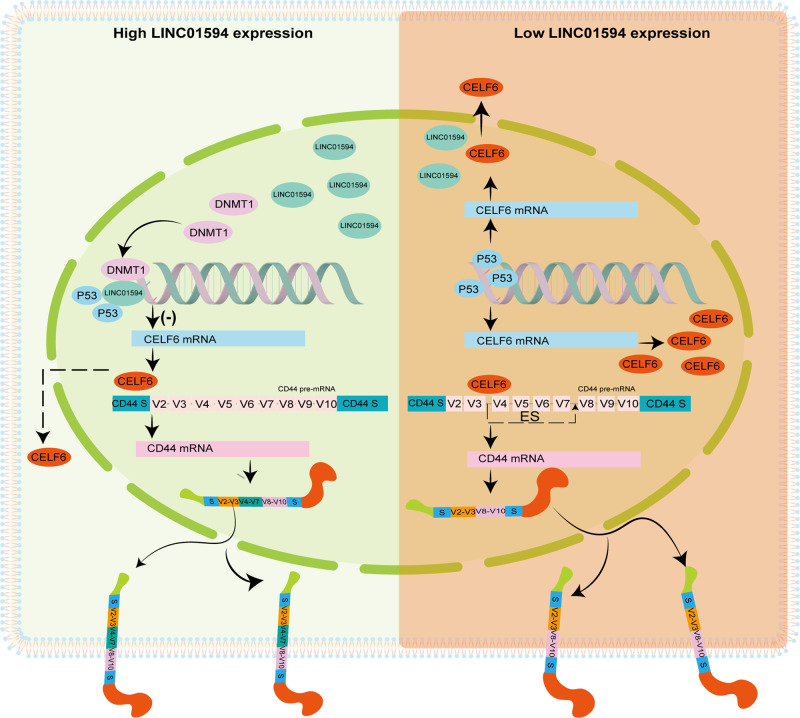
A hypothetical model depicts the roles of LINC01594 in CRC metastasis. LINC01594 is upregulated in CRC and promote CRC metastasis by inhibiting CELF6, which induced the splicing of oncogenic CD44 variants.

Aberrant methylation is associated with progression in several cancers [56]. Eric Batsché et al. confirmed that alternative splicing is influenced by DNA methylation and that splicing disruptions are nearly as commonly responsible for cancer as DNA mutations [57]. We showed that the magnitude of the regulation of alternative splicing events by CELF6 also depends on DNMT1-mediated DNA methylation. We confirmed the different result reported by Liu et al., who found that CELF6 is induced by p53 [19]. Bruna R. Muys et al. reported in 2021 that the p53-induced splicing factor ZMAT3 regulates CD44 alternative splicing [30]. In accordance with the results reported by Bruna R. Muys et al., the present research shows that p53 regulates CD44 alternative splicing through CELF6. Overall, these results indicate that p53 may be closely related to CD44 alternative splicing; on the other hand, over half of CRCs harbor p53 missense mutations, and LINC01594 regulates CELF6 through dual mechanisms, ensuring that the LINC01594/CELF6/CD44 axis is stable in CRCs (Fig. 9).

## Conclusion

In summary, we identified a novel lncRNA, LINC01594, in CRC cells. LINC01594 increases the expression of CD44 V4–V7 through inhibiting of CELF6, a splicing factor, leading to CRC metastasis. These findings provide novel mechanistic insight into CRC metastasis and uncover LINC01594 as a possible target in the treatment of metastatic CRC.

## Supplementary information

Additional file 1

Additional file 2

Additional file 3

Additional file 4

Original Data File

checklist

## Data Availability

All data generated or analyzed during the current study are available from the corresponding author on reasonable request.
