# Supplementary material for: Long noncoding RNA LINC01594 inhibits the CELF6-mediated splicing of oncogenic CD44 variants to promote colorectal cancer metastasis
Source: Cell Death Dis. 2023 Jul 14;14(7):427. doi: 10.1038/s41419-023-05924-8 (PMC10349055; doi:10.1038/s41419-023-05924-8)
Supplement: Supplementary file 1 — Additional file 1 [file 41419_2023_5924_MOESM1_ESM.docx]

**Table S1, Clinical characteristics of 60 CRC samples for qRT-PCR validation.**

| **Characteristics Variable Patients (60) Percentages(%)** | | | |
| --- | --- | --- | --- |
| Age | ＜60 years | 24 | 40 |
|  | ≥60 years | 36 | 60 |
| Gender | Male | 33 | 55 |
|  | Female | 27 | 45 |
| TNM stage | Ⅰ | 10 | 16.7 |
|  | Ⅱ | 20 | 33.3 |
|  | Ⅲ | 24 | 40 |
|  | Ⅳ | 6 | 10 |
| Depth of invasion | T1 | 2 | 3.3 |
|  | T2 | 19 | 31.7 |
|  | T3 | 29 | 48.3 |
|  | T4 | 10 | 16.7 |
| Lymph node metastasis | N0 | 19 | 31.7 |
|  | N1 | 28 | 46.7 |
|  | N2 | 13 | 21.7 |
| Distant metastasis | M0 | 50 | 83.3 |
|  | M1 | 10 | 16.7 |

TNM Staging is referring to the AJCC 8^th^ edition TNM Staging Criteria.

Data are presented as number(%)
