# Supplementary material for: Long noncoding RNA LINC01594 inhibits the CELF6-mediated splicing of oncogenic CD44 variants to promote colorectal cancer metastasis
Source: Cell Death Dis. 2023 Jul 14;14(7):427. doi: 10.1038/s41419-023-05924-8 (PMC10349055; doi:10.1038/s41419-023-05924-8)
Supplement: Supplementary file 2 — Additional file 2 [file 41419_2023_5924_MOESM2_ESM.docx]

**Table S2, siRNA sequences, probes sequences and primer sequences were listed.**

**siRNAs**

LINC01594-si-38: Sense 5’ –GGGUAAAGAUUGAAGGAUAUG–3’

Anti-sense 5’ –UAUCCUUCAAUCUUUACCCAG–3’

LINC01594-si-570: Sense 5’ –CAGACACUGUUACAACAGACA–3’

Anti-sense 5’ –UCUGUUGUAACAGUGUCUGGA–3’

LINC01594-si-147: Sense 5’ –GGAGAGAUAAAAUGUUCUAUU–3’
 Anti-sense 5’ –UAGAACAUUUUAUCUCUCCUU–3’

CELF6-si-1111: Sense 5’- GCAACACGUGGCGGCCUUUTT-3’

Anti-sense 5’- AAAGGCCGCCACGUGUUGCTT-3’

**LINC01594-3’ FAM-5’ FAM probes sequence**

5’-GTGTCTGGAGGGATTCAATGCTTAGTGGATGGC-3’

**LINC01594 ChIRP probes sequence**

5’-CATTTTATCTCTCCTAGTGT-3’

5’-TTGCAAAGCTGAGAACCAGG-3’

5’-TAGTCCTTAGTAGTCTTGTC-3’

5’-TTCAATGCTTAGTGGATGGC-3’

**Primers**

LINC01594 Forward primers: 5’-GTGGTAAGCACTGCAGTAGGA-3’

LINC01594 Reverse primers: 5’- AGCCCAGTACAGGTTTTTGTCT-3’

CELF6 Forward primers: 5’- GCACCACTACGCAGCA-3’

CELF6 Reverse primers: 5’- CGCTTTAGCTGGACCTTGAG -3’

18S Forward primers: 5’- GTAACCCGTTGAACCCCATT-3’

18S Reverse primers: 5’- CCATCCAATCGGTAGTAGCG-3’

GAPDH Forward primers: 5’- GAAGGTGAAGGTCGGAGTC-3’

GAPDH Reverse primers: 5’- GAAGATGGTGATGGGATTTC -3’

CD44 standard isoform Forward primers: 5’-AATCAGATGGACACTCACATGGG-3’

CD44 standard isoform Reverse primers: 5’- TCCCAGCTCCCTGTAATGGTTATG-3’

CD44s Forward primers: 5’-AGAATCCCTGCTACCAGAGACCAA-3’

CD44s Reverse primers: 5’- CCCATGTGAGTGTCCATCTGATT-3’

(CD44s product length: 690bp, CD44v product length > 690bp)

CD44 V4-V7 Forward primers: 5’- CTTTTGACCACACAAAACAGAAC-3’

CD44 V4-V7 Reverse primers: 5’- GGTTGAAGAAATCAGTCCAGGAA-3’

LINC01594-RIP-Forward primer-1: 5’- AGAATGATCAGGGAAGACATGACT-3’

LINC01594-RIP-Reverse primer-1: 5’- AAGTGAGGGCCTGAGTGAAT-3’

LINC01594-RIP-Forward primer-2: 5’- GTGGCAGTATGTTAGAGAAGACT-3’

LINC01594-RIP-Reverse primer-2: 5’- CTAATGTTCAGTAAATGTCAGCTG-3’

LINC01594-RIP-Forward primer-3: 5’- CAATGTGGTAAGCACTGCAGT-3’

LINC01594-RIP-Reverse primer-3: 5’- GTAGCCCAGTACAGGTTTTTGTCT-3’

LINC01594-RIP-Forward primer-4: 5’- TCTTGGCAGAGGAAATGCC-3’

LINC01594-RIP-Reverse primer-4: 5’- GTAGTCTTGTCTTGAAAATGATATTTCA-3’

LINC01594-RIP-Forward primer-5: 5’- GGACTAATCTTCATCATCATCATCA-3’

LINC01594-RIP-Reverse primer-5: 5’- AGGGATTCAATGCTTAGTGGATGG-3’

CELF6 MSP M primer Forward primers: 5’-TTTATTTTCGTAGGAAACGTATTAGC-3’

CELF6 MSP M primer Reverse primers: 5’- CTACTCGCCATAACAACCGA -3’

CELF6 MSP U primer Forward primers: 5’- ATTTTTGTAGGAAATGTATTAGTGT -3’

CELF6 MSP U primer Reverse primers: 5’- CTCCTACTCACCATAACAACCAAA -3

CELF6 CpG island ChIP Forward primers: 5’- TCCGAGGGACTGGCTCG-3’

CELF6 CpG island ChIP Reverse primers: 5’- GGCTCACGCGCTCCTG -3’

CELF6 promoter ChIP Forward primers 1: 5’- GATGATTCCAGTTGCTACTGCTGG-3’

CELF6 promoter ChIP Reverse primers 1: 5’- GGGATGACAGGTGCACGCTA -3’

CELF6 promoter ChIP Forward primers 2: 5’- AGGCTTAGGTGGGAGGATC -3’

CELF6 promoter ChIP Reverse primers 2: 5’- CCACCCCAACATTTGTTGGG -3’

CELF6 promoter ChIP Forward primers 3: 5’- CATCCTCGACCCTCAGAACC -3’

CELF6 promoter ChIP Reverse primers 3: 5’- GAGTGAGGGAACTTAGCCGT -3’

CELF6 promoter ChIP Forward primers 4: 5’- TAAGGCTGCAAGGGAGACGA -3’

CELF6 promoter ChIP Reverse primers 4: 5’- GTCGGGGCTAATCCCCACC -3’

CELF6 promoter ChIP Forward primers 5: 5’- CATCACGCATGACGAAGCTG -3’

CELF6 promoter ChIP Reverse primers 5: 5’- CGCCAATCAGAGTGGGAGTT -3’
