# Supplementary material for: Long noncoding RNA LINC01594 inhibits the CELF6-mediated splicing of oncogenic CD44 variants to promote colorectal cancer metastasis
Source: Cell Death Dis. 2023 Jul 14;14(7):427. doi: 10.1038/s41419-023-05924-8 (PMC10349055; doi:10.1038/s41419-023-05924-8)
Supplement: Supplementary file 3 — Additional file 3 [file 41419_2023_5924_MOESM3_ESM.docx]

**Table S3 Relationship between LINC01594 expression and clinicopathological features in TMAs specimens.**

| **Variables Cases LINC01594 expression (n=73) *p^a^*** | | | | |
| --- | --- | --- | --- | --- |
| **Low (n=37) High (n=36)** | | | | |
| Age |  |  |  | 0.8042 |
| ＜60 | 24 | 13 | 11 |  |
| ≥60 | 49 | 24 | 25 |  |
| Gender |  |  |  | 0.0618 |
| Female | 39 | 24 | 15 |  |
| Male | 34 | 13 | 21 |  |
| Tumor diameter (cm) |  |  |  | 0.3617 |
| ≤5cm | 29 | 17 | 12 |  |
| ＞5cm | 44 | 20 | 22 |  |
| Depth of invasion |  |  |  | **0.0456** |
| T1/T2 | 41 | 25 | 16 |  |
| T3/T4 | 32 | 12 | 20 |  |
| Lymph node metastasis |  |  |  | **0.0263** |
| N0 | 44 | 30 | 14 |  |
| N1/N2 | 29 | 7 | 22 |  |
| Distant metastasis |  |  |  | **0.0003** |
| M0 | 53 | 30 | 23 |  |
| M1 | 20 | 7 | 13 |  |
| TNM stage |  |  |  | **0.0468** |
| Ⅰ/Ⅱ | 37 | 23 | 14 |  |
| Ⅲ/Ⅳ | 36 | 14 | 22 |  |
| Differentiation |  |  |  | 0.4656 |
| Middle/High | 34 | 16 | 18 |  |
| Poor | 39 | 21 | 18 |  |

*p*-value ^a^ was measured by Pearson’s Chi-Squared test.

**Table S4, Relationship between LINC01594 expression and clinicopathological features in fresh-frozen specimens.**

| **Variables Cases LINC01594 epression (n=60) *p*** | | | | |
| --- | --- | --- | --- | --- |
| **Low (n=30) High (n=30)** | | | | |
| Age (years) |  |  |  | 0.4296 |
| ＜60 | 24 | 14 | 10 |  |
| ≥60 | 36 | 16 | 20 |  |
| Gender |  |  |  | 0.4416 |
| Female | 33 | 13 | 20 |  |
| Male | 27 | 17 | 10 |  |
| Tumor diameter (cm) |  |  |  | 0.7866 |
| ≤5 | 39 | 20 | 19 |  |
| ＞5 | 21 | 10 | 11 |  |
| Depth of invasion |  |  |  | **0.0029** |
| T1/T2 | 21 | 16 | 5 |  |
| T3/T4 | 39 | 14 | 25 |  |
| Lymph node metastasis |  |  |  | **0.0023** |
| N0 | 19 | 15 | 4 |  |
| N1/N2 | 41 | 15 | 26 |  |
| Distant metastasis |  |  |  | **0.0377** |
| M0 | 50 | 28 | 22 |  |
| M1 | 10 | 2 | 8 |  |
| TNM stage |  |  |  | **0.0389** |
| Ⅰ | 10 | 8 | 2 |  |
| Ⅱ | 20 | 11 | 9 |  |
| Ⅲ | 24 | 10 | 14 |  |
| Ⅳ | 6 | 1 | 5 |  |
| Differentiation |  |  |  | 0.4257 |
| Middle/High | 23 | 10 | 13 |  |
| Poor | 37 | 20 | 17 |  |

p-value ^a^ was measured by Pearson’s Chi-Squared test.
