# Supplementary material for: Long noncoding RNA LINC01594 inhibits the CELF6-mediated splicing of oncogenic CD44 variants to promote colorectal cancer metastasis
Source: Cell Death Dis. 2023 Jul 14;14(7):427. doi: 10.1038/s41419-023-05924-8 (PMC10349055; doi:10.1038/s41419-023-05924-8)
Supplement: Supplementary file 4 — Additional file 4 [file 41419_2023_5924_MOESM4_ESM.docx]

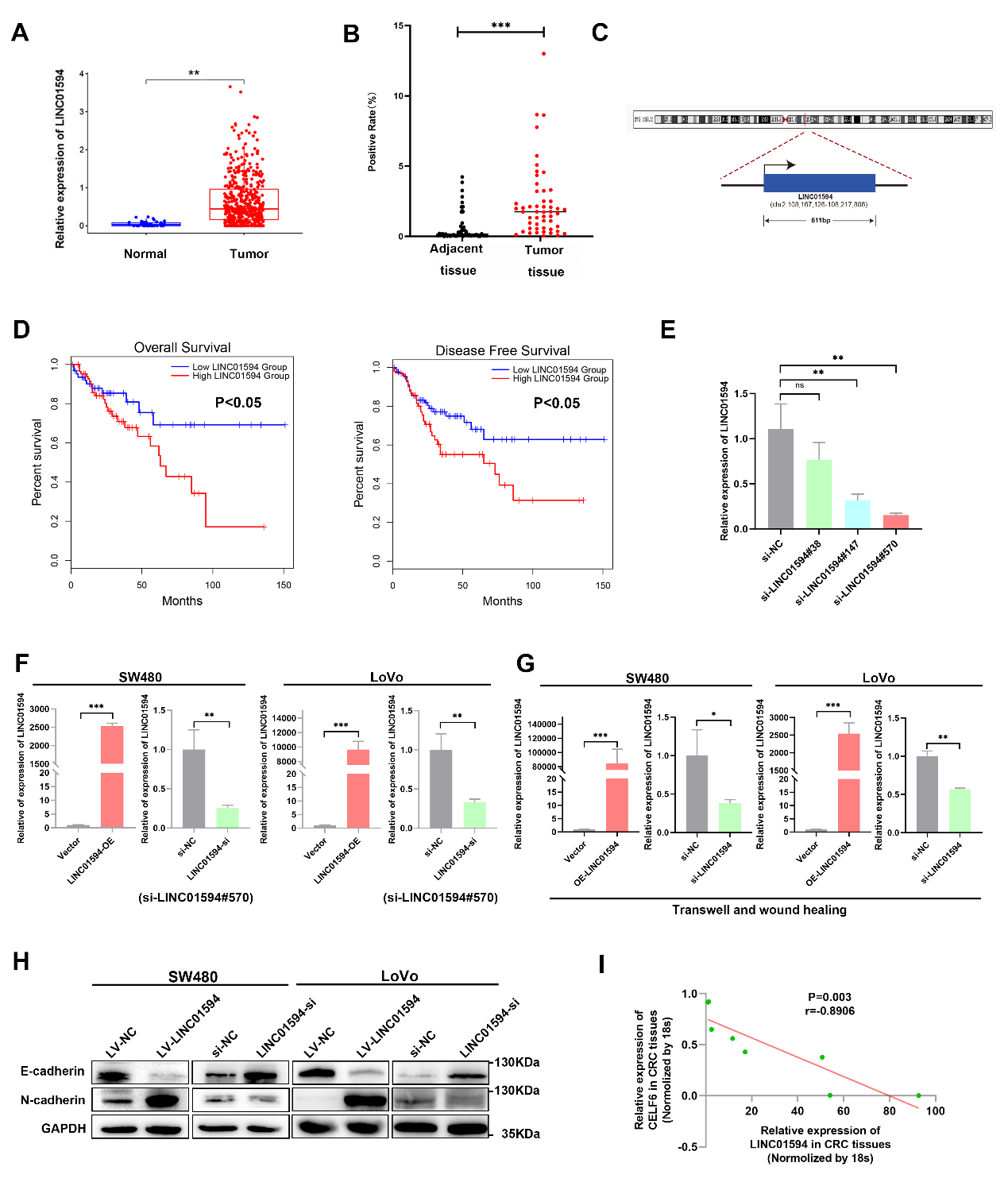


Figure S1. A, LINC01594 expression in TCGA CRC RNA-seq dataset (P<0.05). B, FISH was used to determine the expression of LINC01594 in CRC TMAs and statistical data are shown. C, Data from NCBI showed the characteristic of LINC01594 genomic locus and full length. D, Kaplan-Meier overall survival and disease-free survival curves of LINC01594 in TCGA database. E, qRT-PCR was used to detect three siRNAs, si-LINC01594#38, si-LINC01594#147, si-LINC01594#570, and the knockdown efficiency was detected. (n=3). **p<0.01. F, Overexpression and knockdown efficiency of LINC01594 were detected by qRT-PCR in SW480 cells and LoVo cells. (n=3). **p<0.01, ***p<0.001. G, qRT-PCR was used to detect the LINC01594 expression level in Transwell and wound healing assay. (n=3). * p<0.05, **p<0.01, ***p<0.001. H, The expression of E-Cadherin and N-Cadherin in CRC cells with knockdown or overexprerssion of LINC01594 were determained by western blot. I, qRT-PCR was used to detect CELF6 and LINC01594 co-expression in CRC tissues, and correlation analysis confirmed that LINC01594 negatively associated with CELF6.


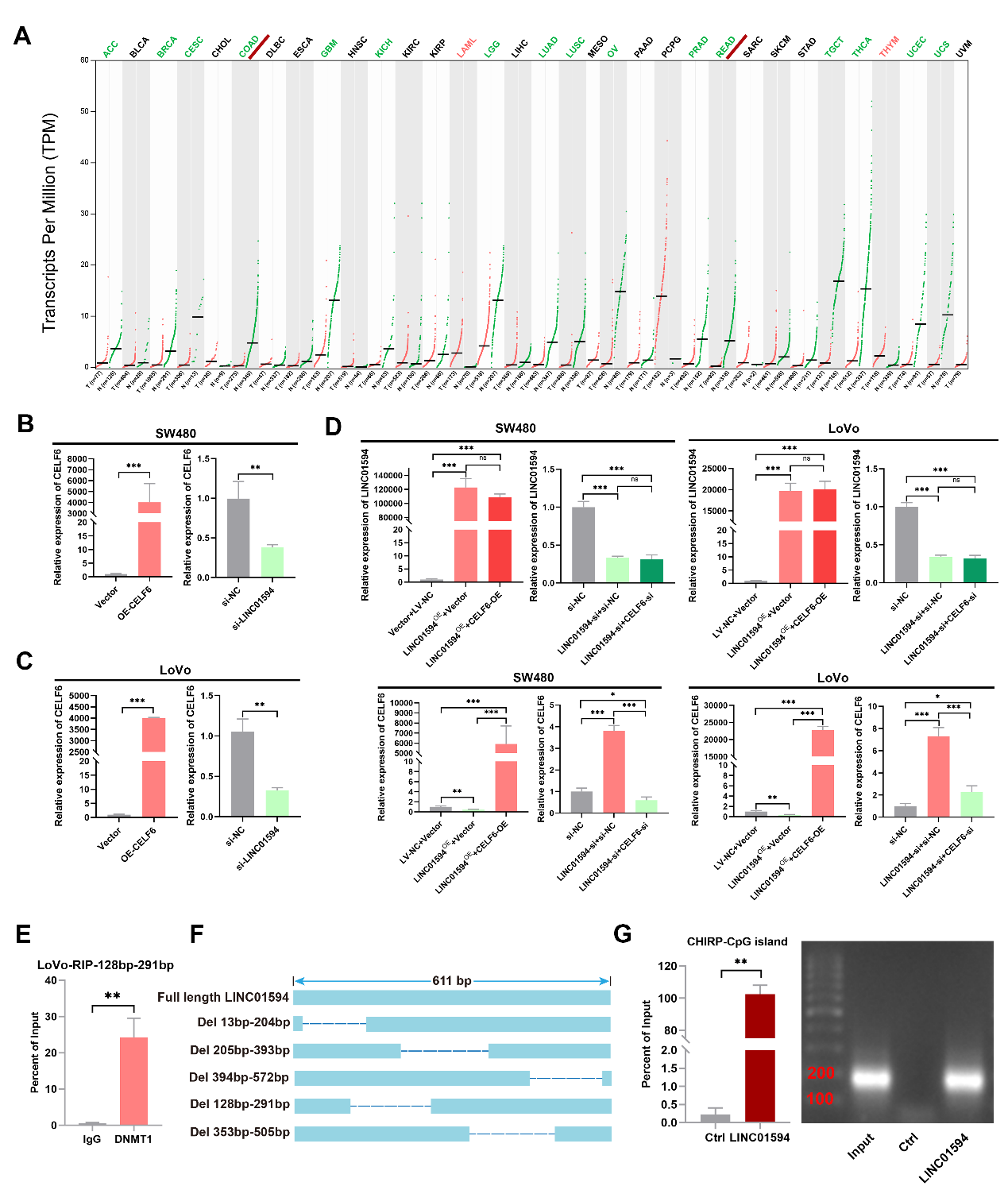


Figure S2. A, CELF6 was significantly downregulated in multiple tumors. B-C, the overexpression or knockdown efficiency of CELF6 was determained by qRT-PCR in Tanswell assay. D, the expression level of LINC01594 and CELF6 were detected by qRT-PCR in Transwell and wound healing assay. E, RIP-qPCR verified that LINC01594 binds with DNMT1 in the 128-291 bp region in both LoVo cells. (n=3). **p<0.01. F , LINC01594 truncations were designed that deleted 13-204 bp; 205-393 bp; 394-572 bp; 128-291 bp; 253-505 bp. G, DNA enrichment in ChIRP experiments with control and LINC01594 probes determined by qPCR and calculated as percentage of Input with the indicated primer sets. (n=3). **p<0.01.
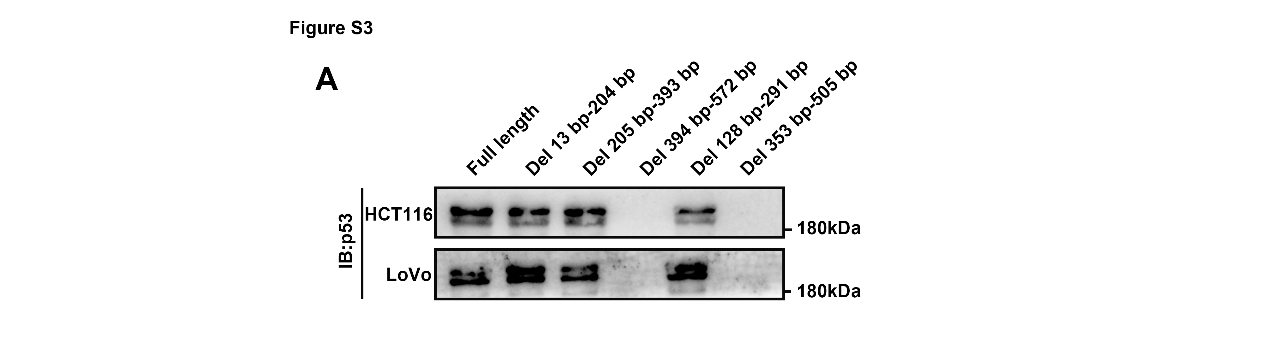


Figure S3. A, Biotinylated LINC01594 with deletions were used in RNA-pulldown assay.


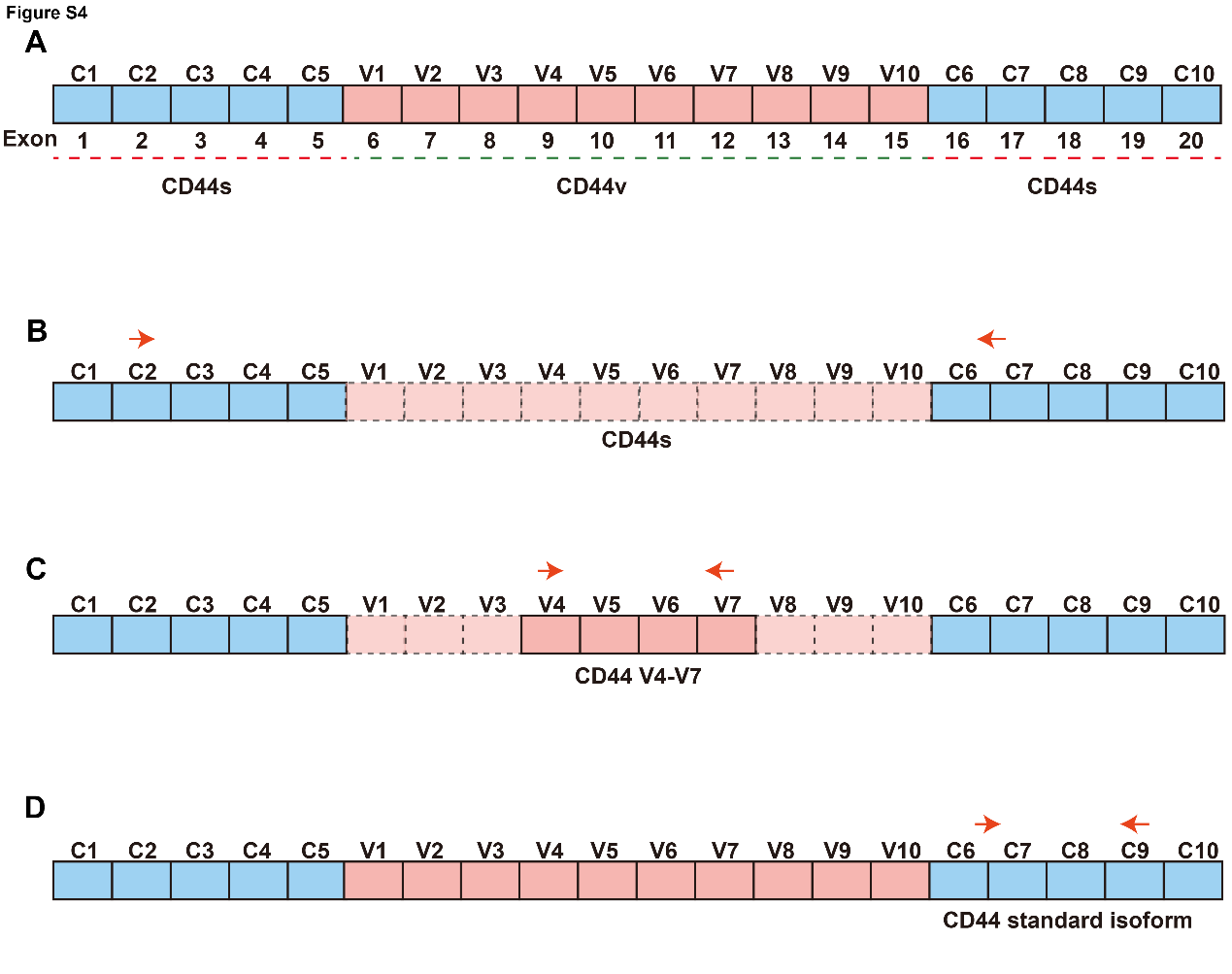


Figure S4. A, CD44 full length isoform was showed. B, To determine the CD44s the forward primer was designed on exon 2 and reverse on exon 15 and exon 16 of CD44 mRNA, the CD44s was 690 bp. C, The forward primers of CD44 V4-V7 was designed on variant exon 4 and reverse primers was designed on variant exon 7. D, The CD44 standard isoform primers was designed to detect exon 15 to exon 18.
