# Supplementary figures and images for: Long noncoding RNA LINC01594 inhibits the CELF6-mediated splicing of oncogenic CD44 variants to promote colorectal cancer metastasis
Source: Cell Death Dis. 2023 Jul 14;14(7):427. doi: 10.1038/s41419-023-05924-8 (PMC10349055; doi:10.1038/s41419-023-05924-8)

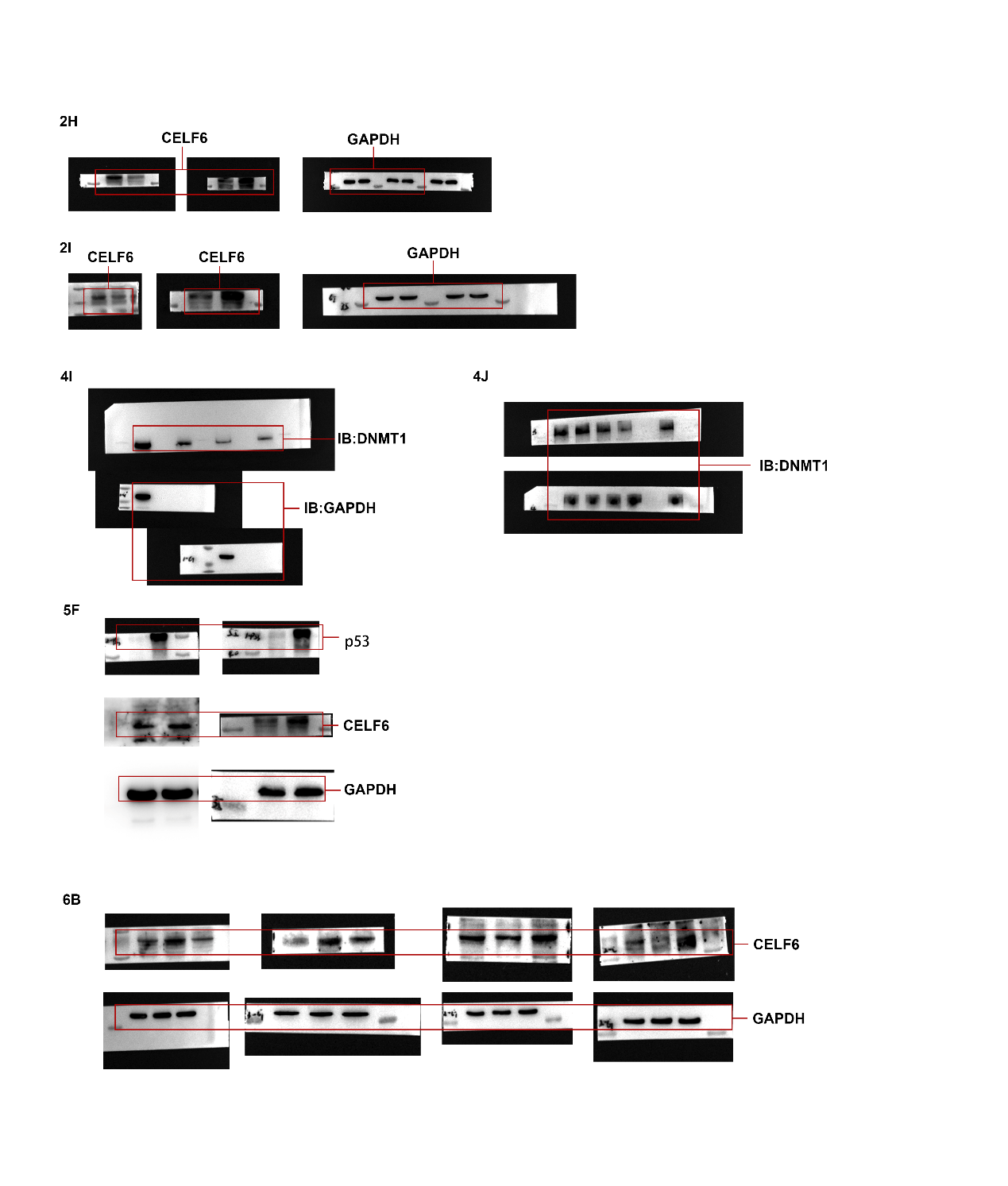


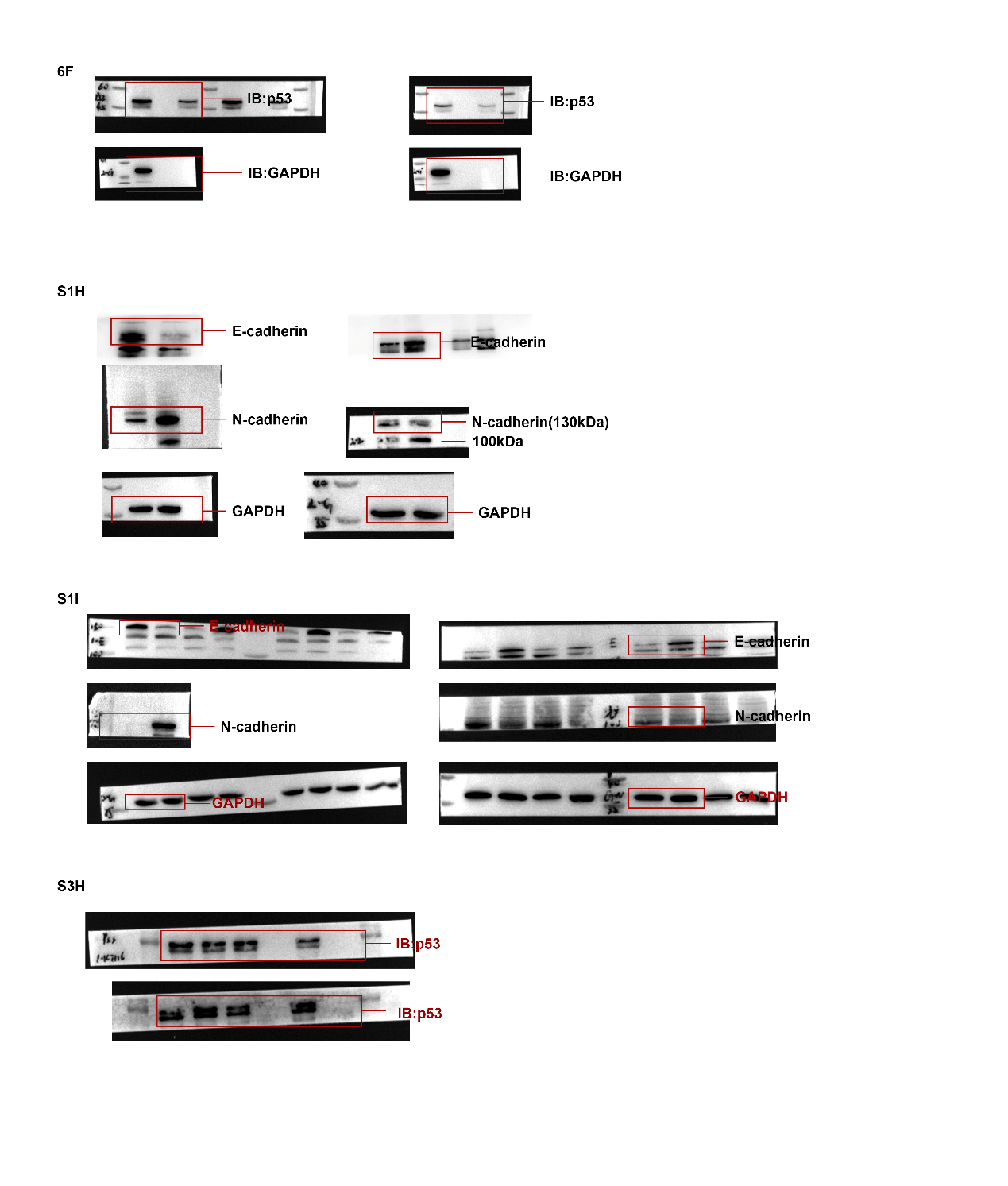

Supplement: Supplementary file 5 — Original Data File [file 41419_2023_5924_MOESM5_ESM.docx]
